# Isotropic Gels of Cellulose Nanocrystals Grafted with Dialkyl Groups: Influence of Surface Group Topology from Nonlinear Oscillatory Shear

**DOI:** 10.1021/acs.langmuir.3c00210

**Published:** 2023-04-25

**Authors:** Sylwia Wojno, Amit Kumar Sonker, Jelka Feldhusen, Gunnar Westman, Roland Kádár

**Affiliations:** † Department of Industrial and Materials Science, Division of Engineering Materials, Chalmers University of Technology, SE-412 96 Gothenburg, Sweden; ‡ Department of Chemistry and Chemical Engineering, Division of Chemistry and Biochemistry, Chalmers University of Technology, SE-412 96 Gothenburg, Sweden; § Wallenberg Wood Science Center (WWSC), Chalmers, SE-412 96 Gothenburg, Sweden

## Abstract

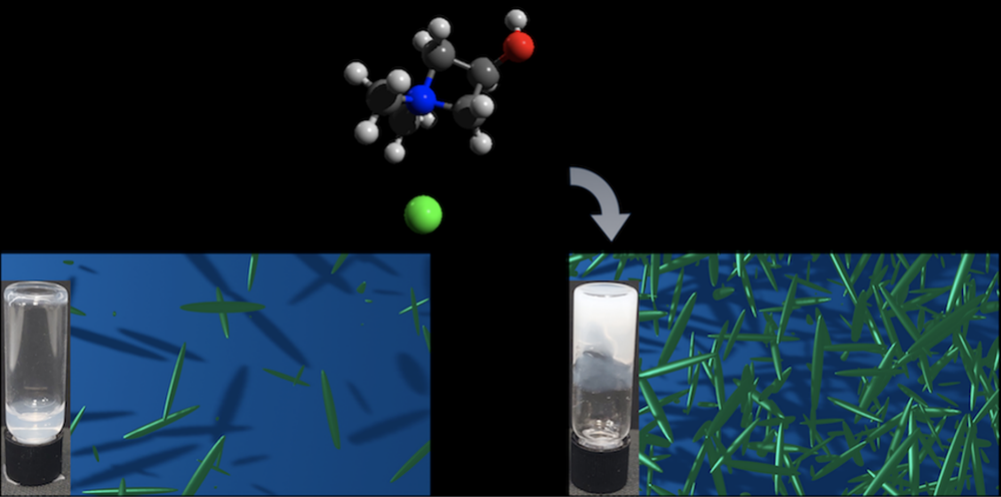

Attractive (non-self-assembling) aqueous cellulose nanocrystal (CNC) suspensions were topologically tailored into isotropic gels through the surface grafting of dialkyl groups. We thus focus on the influence of CNC concentration, including for pristine CNC, surface linker branching, branching degree, and the influence of side group size and branch-on-branch surface-grafted groups. The resulting mobility and strength of interaction in particle–particle interaction mediated by the surface groups was investigated from a rheological point of view. The emphasis is on nonlinear material parameters from Fourier-transform rheology and stress decomposition analysis. The results show that nonlinear material parameters are more sensitive than linear viscoelastic parameters to the onset of weakly interconnected networks in pristine CNC isotropic suspensions. All surface-modified CNC suspensions resulted in isotropic gels. The nonlinear material parameters were found to be broadly sensitive to CNC concentration, branching, degree of branching and surface-grafted linkers’ length. However, the length of the grafted chains and the degree of branching were the primary factors influencing the nonlinear material response. Furthermore, the results showed evidence of two strain amplitude ranges with distinct nonlinear signatures that could be attributed to the disruption of weak network connection points and to distortions of more dense (aggregate) network regions, respectively.

## Introduction

Cellulose is an abundant natural resource on earth, present in wood, tunicates, algae, and bacteria.^1^ Through acid-catalyzed hydrolysis that removes the amorphous part of cellulose nanofibrils, cellulose nanocrystals (CNCs) can be obtained.^2^ As a result, CNCs are rod-like nanoparticles containing only crystalline domains. In addition to availability from renewable resources, CNCs are biodegradable and nontoxic.^3^ CNCs are a nanomaterial of high contemporary interest with significant potential for new applications.^4−7^ As rheological modifiers, for films, biocomposites, etc., understanding and controlling interparticle interaction is of paramount importance for obtaining materials with favorable performance. Two of the most common means for structuring CNC-based materials involve surface modification of CNCs and/or through the effects of an imposed flow.^4,8^

### CNC Surface Modification

The surface charge and particle size of the selected CNC depend on the hydrolysis method or source of cellulose. HCl hydrolysis gives neutral CNC, whereas sulfuric acid hydrolysis gives sulfate half-ester-decorated CNC (CNC-OSO_3_H). Functionalization of the CNCs with different components can significantly change their properties, such as thermal stability and mechanical properties. Therefore, surface chemical modifications face many challenges and at the same time provide more opportunities for new applications.^9−16^ Various surface modification strategies have been proposed including esterification, etherification, click chemistry, and polymer grafting.^17,18^ For the surface modification of the sulfate groups, it has been reported that azetidinium salts can be used for conjugation.^19,20^ Sahlin et al.^20^ have investigated a number of modified CNC suspensions at different concentrations. Surface loading may also have an effect on the pristine CNC since the proximity of the sulfate groups most likely affects the hydrogen network between different sulfate groups and the solvent, water in this case.

### Rheology of Attractive CNC Dispersions

Over the past decades, assessing rheological properties has become an integral part of characterizing different forms of cellulose. Physicochemical properties of particles affect the rheological behavior of CNC suspensions.^21^ In general, depending on whether attractive or repulsive forces between CNCs dominate, aqueous CNC suspensions either form isotropic, biphasic (containing both liquid crystalline and isotropic domains), liquid crystalline, and repulsive glasses so-called phases or form isotropic suspensions, isotropic (attractive) gels.^22^ We briefly note that the assigning of the term “gel” can vary between different nomenclatures, and here, we refer to it strictly from a rheological perspective.^23^ While in the case of nonself-assembling CNC systems their photonic properties are lost, they are important for applications as rheology modifiers and fillers in nanocomposites.^24^

Most rheological studies on CNC suspensions focus on the linear viscoelastic behavior in dynamic tests, with nonlinear conditions achieved typically through steady shear tests and on self-assembling CNC systems. Attractive CNC suspensions have been investigated by Sahlin et al.,^20^ who evaluated the rheological properties of dispersions based on two solid contents of pristine and surface-modified CNCs. Stokes et al.^25^ studied the impact of pH and salinity on the rheological properties, especially steady shear viscosity. Low pH decreased the viscosity, while for pH > 12, the viscosity dramatically increased. A similar observation with a higher amount of NaCl was done by Danesh et al.^26^ They were able to distinguish two yield stresses, where the first one was due to the yielding and flow of clusters, while the second one was due to the breakage of clusters to small flocs and individual particles. Using a different experimental approach, Pignon et al.^27^ studied breakup and buildup mechanisms of CNC suspensions under shear and upon relaxation. In turn, our previous study on surface-modified CNC suspensions was focused on examining the influence of the surface linkages on the self-assembly of the suspensions using rheology combined with polarized light imaging (rheo-PLI)^28^ with supporting of molecular dynamics simulations.

Nonlinear oscillatory shear analysis via Fourier-transform rheology (FTrheology), stress decomposition, and other nonlinear analysis techniques has gained increasing attention as a rheological testing framework with increased sensitivity and new (nonlinear) material rheological parameters potentially revealing material response features which are not observable in linear viscoelastic measurements.^29^ Transferring techniques from NMR spectroscopy to oscillatory rheometry helped develop the experimental methodology for FTrheology^30^ by increasing the sensitivity of the torque transducer. Recently, Abbasi-Moud et al.^31^ have investigated the nonlinear viscoelastic behavior of CNCs in the presence of sodium chloride using stress decomposition methods. They used nonlinear techniques to correlate the macromechanical viscoelastic response of the CNC/salt aqueous systems to structural changes as a response to strain amplitude. A strong dependence of the nonlinear response of the materials to salt concentration, CNC concentration, and frequency of deformation was shown. Our previous work focused on investigating the nonlinear rheological characterization and phase transitions of self-assembling CNC suspensions at different concentrations.^32^ Nonlinear parameters from FTrheology and stress-decomposition analysis effectively distinguished the isotropic, biphasic, and liquid crystalline phases. Subsequently, we extended the study of the relevance of nonlinear material parameters to include a decoupling between phase transitions and percolation and gel point through desulfation.^23^

In contrast to our previous work, we concentrate here on attractive CNC systems, i.e., suspensions that do not self-assemble into liquid crystalline domains. Furthermore, the CNCs are further surface modified by grafted branched alkyl groups. The modified CNC suspensions were synthesized by conjugating azetidinium salts to sulfate half-ester available on the CNC surface. We analyze two sets of samples: pristine suspensions with increasing CNC concentration and surface-modified CNC suspensions at two concentrations. Our hypothesis is that by comparing the linear and nonlinear material response of the two sets of data, we can elucidate the assembly behavior of pristine attractive CNC suspensions and what are the contributions of surface modifications to the nonlinear material response as determined from Fourier-transform rheology and stress decomposition analysis.

## Materials and Methods

### CNC Preparation and Surface Modification

Suspensions of pristine CNC-OSO_3_H were prepared by acid hydrolysis with sulfuric acid, H_2_SO_4_, of microcrystalline cellulose (MCC) using the procedure described by Hasani et al.^33^ The obtained CNC-OSO_3_H had a sulfate content of 330 μmol/g. The concentration of this stock suspension was 5.7 wt %. Deionized water (Millipore Milli-Q purification system) was used to dilute the stock suspension and obtain 6 different concentrations of pristine CNC (pCNC) suspensions: 1, 1.5, 2, 3, 4, and 5 wt % (weight %).

#### Sulfate Half-Ester Content

The sulfate half-ester content of each CNC sample was determined through potentiometric titration (Figure SI1). A 20 mL amount of 0.5 wt % CNC suspensions was subjected to titration to determine sulfate half esters. The samples were titrated against 0.1 M NaOH.

#### CNC Modification with Azetidinium Salts

Five different azetidinium salts from their respective open-form structure and corresponding amines, namely, 1-hydroxy-1-nonyl-1-propylazetidin-1-ium chloride, 3-hydroxy-1-methyl-1-undecylazetidin-1-ium chloride, 1-hexyl-3-hydroxy-1-undecylazetidin-1-ium chloride, 1-hexyl-3-hydroxy-1-undecylazetidin-1-ium chloride, and 1-(2-ethylhexyl)-3-hydroxy-1-undecylazetidin-1-ium chloride with respective nomenclature C_9_-N-C_3_-Prop-2-OH, C_11_-N-C_1_-Prop-2-OH, C_11_-N-C_3_-Prop-2-OH, C_11_-N-C_6_-Prop-2-OH, and C_11_N-C_6(2Et)_-Prop-2-OH, were synthesized to modify cellulose nanocrystals surface by conjugation to available sulfate half-ester groups (−OSO_3_H). See Supporting Information for detailed synthesis and characterization (^1^H NMR and ^13^C NMR) of dialkyl amines and azetidinium salts. We would like to emphasize that we use shortened forms in the text for brevity. The ratio of azetidinium salt to CNC dispersion was taken in accordance with the surface sulfate content as the same mole (1:1) equivalent. The conjugation of azetidinium salt to sulfate half-ester was performed by heating the mixture at 90 °C for 4 h. When the reaction had been completed, it was cooled to room temperature. The reaction mixture was transferred into dialysis tubing (Spectra/Por molecular porous membrane tubing, MWCO 12–14 kDa) and dialyzed against deionized water for 48 h to remove unreacted azetidinium reagent. Deionized water was replaced approximately every 12 h, and conductivity measurements were taken before and after unless stable conductivity of less than 5 μS was shown. Suspensions containing 1.5 and 3 wt % CNC-OSO_3_H were prepared for all modified salts.

**Figure 1:**
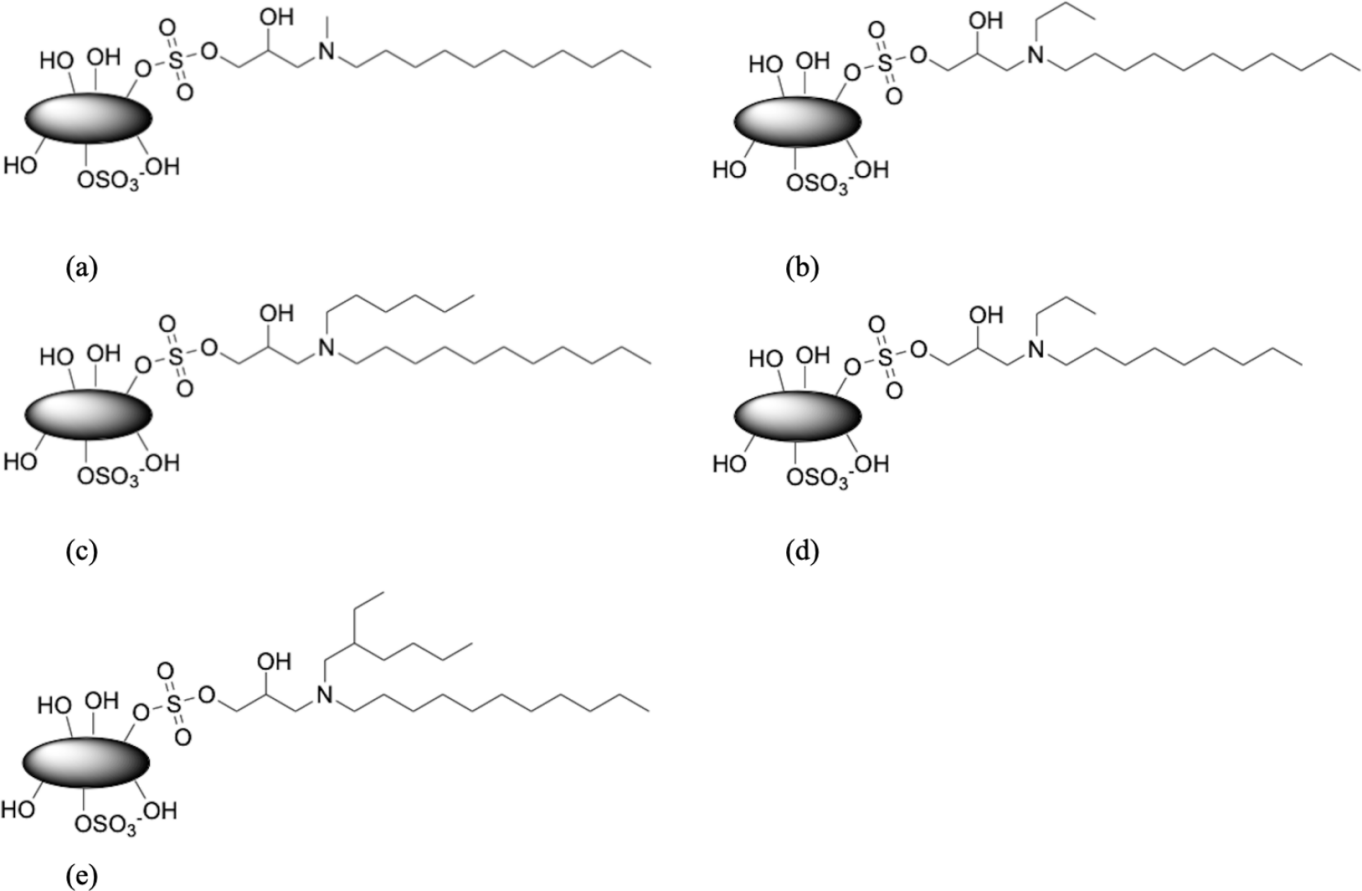
Overview of CNC surface modifications investigated in this study (a–e).

The modified CNC with different *N*-dialkyl groups are listed below using their notations of molecular structures with a schematic overview presented in Figure 1:

(a) C_11_-N-C_1_-Prop-2-OH-CNC,
(b) C_11_-N-C_3_-Prop-2-OH-CNC,
(c) C_11_-N-C_6_-Prop-2-OH-CNC,
(d) C_9_-N-C_3_-Prop-2-OH-CNC,
(e) C_11_-N-C_6(2Et)_-Prop-2-OH-CNC.

Thus, from Figure 1a–c, a generalized structure of C_11_-N-C_*m*_-Prop-2-OH-CNC was designed to emphasize the influence of linker topology, varying from linear to branched with *m* = 1, 3, and 6. Through Figure 1d, the size of the branched topology can also be probed (C_9_-N-C_3_-Prop-2-OH-CNC), while in Figure 1e, the branch-on-branch structures were designed to create a branched structure with less mobility (C_11_-N-C_6(2Et)_-Prop-2-OH-CNC).

Prior to rheological testing, each suspension was stirred for 15 min using a Bransonic Ultrasonic Bath 221 (220 V, 285 W; Brookfield, CT, US) to prevent precipitation. The rheological behavior of CNC suspensions depends significantly on the pretesting sample preparation protocols.^1,34^

### Rheological Characterization

Linear and nonlinear oscillatory shear measurements were performed on an Anton Paar MCR 702 TwinDrive rheometer (Graz, Austria) in strain-controlled mode (separate mode transducer). A parallel plate geometry of 2*R* = 50 mm in diameter with a gap of 1 mm was used. All experiments were conducted at 23 °C. Before measurements, each sample was allowed to relax for 300 s after moving to the gap position (1 mm). Strain sweep measurements were performed within a strain amplitude range of 0.01% to 1500% at the following angular frequencies: 0.6, 1, 2, and 4 rad/s. Frequency sweep tests were performed between 600 and 0.01 rad/s at an imposed strain amplitude of 0.3%. The strain amplitude was selected based on the strain sweep tests so that it was in the linear viscoelastic region. The nonlinear data analysis of the shear stress output signal was performed in the framework of Fourier-transform rheology and stress decomposition analysis. The nonlinear material parameters analyzed in this study, the third relative higher harmonic, *I*_3/1_, and strain-stiffening *S* and shear-thickening *T* ratios were determined using the procedures outlined in Wojno et al.^23,32^ and are described in detail elsewhere.^29,30,35,36^

#### NMR

Nuclear magnetic resonance (NMR) spectroscopy analysis was used to evaluate the structure of the synthesized molecules. ^1^H NMR was done for all samples and ^13^C NMR when necessary. Samples for NMR were prepared by dissolving the salts in 0.7 mL of CDCl_3_ or DMSO in an NMR tube. NMR analysis was conducted with a Varian 400-MR (Agilent). Spectra were analyzed with a MestReNova 11 (Mestrelab S.L, Santiago de Compostela, Spain).

### Zeta Potential

Zeta potential measurements of CNC suspensions were performed using a Zetasizer Nano ZS (Malvern Instruments, UK). CNC suspensions were diluted to 0.01 wt %. A NaCl concentration of 1 mM was added to each sample to compress the electrical double layer.^37,38^ All measurements were conducted at 25 °C with a stabilization time of 120 s and repeated 6 times, and the average value was reported.

#### ATR-FTIR

Fourier-transform infrared spectroscopy (FTIR) was performed on the CNC films using a PerkinElmer Spectrum One instrument (PerkinElmer). The different measurements were done with the attenuated total reflectance (ATR) technique, and the spectra were recorded between 4000 and 400 cm^–1^, with 32 scans being collected with a resolution of 2 cm^–1^.

#### TGA

Thermal gravimetric analysis (TGA) was used to determine the thermal degradation onset temperatures and the residual chars of the CNC films. Analyses were done on a TGA/DSC 3+ Star system (Mettler Toledo, Greifensee Switzerland). Between 2 and 5 mg of each film was placed in a 100 μL aluminum pan subjected to a heating ramp from 25 to 500 °C at a rate of 10 °C/min under a 20 mL/min flow of nitrogen.

## Results and Discussion

### Evaluation of the CNC Surface Modifications

**Figure 2:**
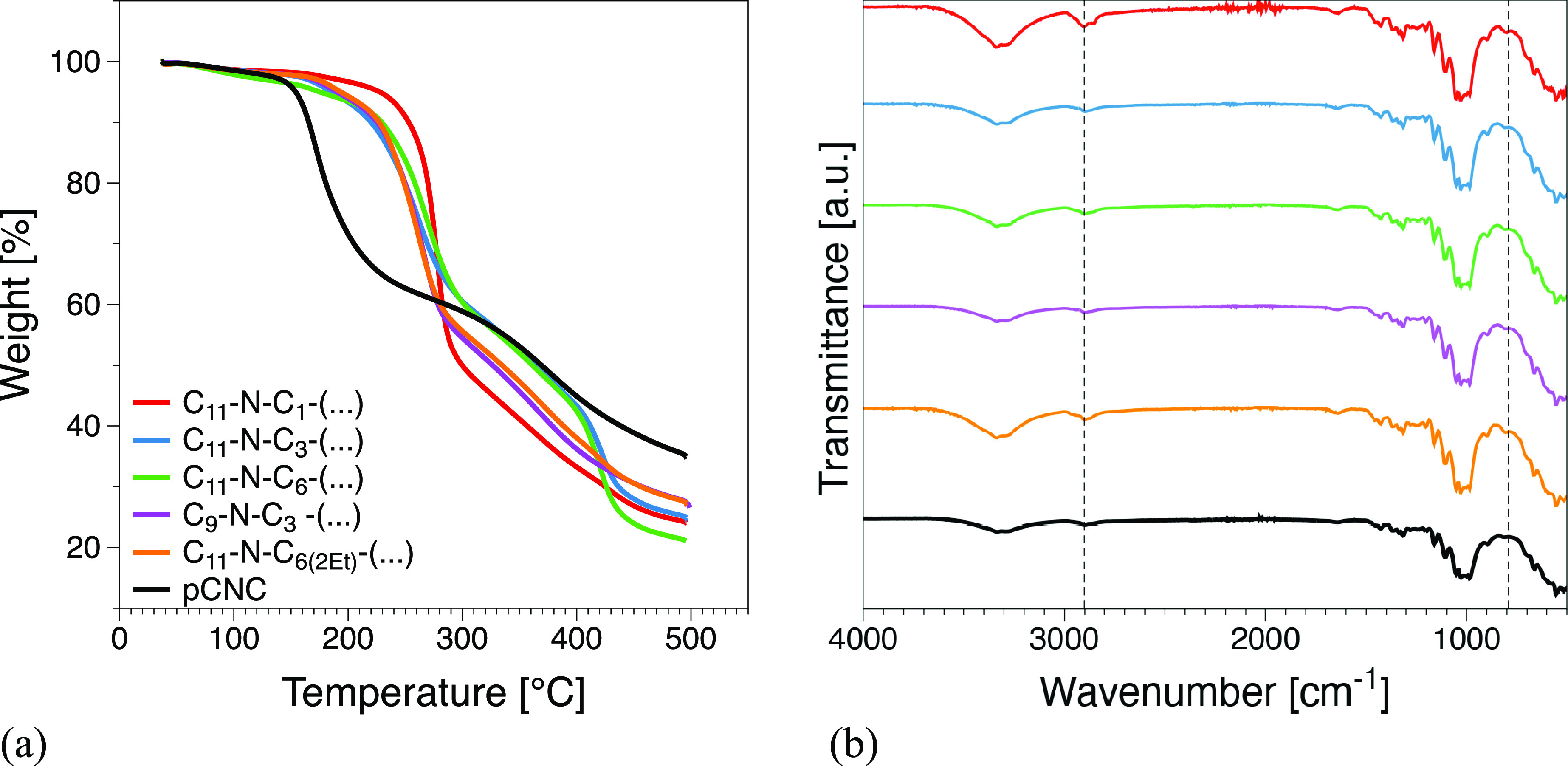
(a) Thermogravimetric curves showing the thermal degradation of investigated materials. (b) Full range FTIR spectra of pristine CNC and modified CNC samples.

The degradation onset temperature, residual char, FTIR, and zeta potential for pristine CNC and modified CNC suspensions are compiled in Table SI1. Thermal degradation curves (TGA) are presented in Figure 2a. In each case, the thermal stability of the modified CNC increased considerably. The degradation onset temperatures of the pristine CNC were 145 and ∼185 °C for the modified CNC samples. Similar results on surface-modified CNCs have been described by Forsgren et al.^24^ A small loss below 150 °C can correspond to the evaporation of hard bound water. Furthermore, two typical mass loss regions can be observed on the TGA curves, both corresponding to a different degradation reaction. The first region is due to cellulose pyrolysis in the presence of hydrogen sulfate which acts as a catalyst. Depolymerization and dehydration of glycosyl units occur since the sulfate groups are present in the hydrogen form. At higher temperatures, the second region is more gradual and corresponds to oxidation and breakdown of the charred residue resulting in low-weight gaseous products. Upon surface conjugation of the dialkyl carbonates, the sulfate hydrogen groups are replaced by the alkyl group, and as a consequence, the hydrolysis degradation reaction is inhibited. Therefore, the increase in degradation onset temperatures of the modified CNC samples confirms the successful surface modification. All CNC systems had an average zeta potential close to stable colloidal suspensions. Bhattacharjee^39^ has estimated that values of >30 mV indicate highly stable CNC colloids in the presence of NaCl. The high value of sulfate content affects on the colloidal stability in the CNC suspensions. Therefore, values around 30 mV are expected.

FTIR full range spectra for pCNC and modified CNC samples are presented in Figure 2b. All six samples gave the expected signal for the presence of cellulose.^40^ A broad band occurs between 3600 and 3000 cm^–1^ corresponding to the −OH groups on cellulose. All samples show a peak at 3000–2900 cm^–1^ due to aliphatic C–H bonds on cellulose and grafted propyl-2-hydroxy-dialkyl chains. The peak at 901 cm^–1^ corresponds to the C–O–C bond between each glucose unit, while the peak at 1640 cm^–1^ corresponds to absorbed water on the cellulose. In addition to other regions, a peak at 814 cm^–1^ corresponds to the C–O–S due to the presence of sulfate half-ester and diester^41^ (Figure SI2a). It is expected that upon surface grafting with azetidinium salts, the peak at 2900 and 814 cm^–1^ must change as there is alkyl branching occurring through the half sulfate esters group on CNC surfaces. Since modified samples have an increased number of CH_2_ groups, they exhibit a stronger peak at approximately 2850 cm^–1^ as expected. Moreover, a small shift from 814 to 808 cm^–1^ in the C–O–S region is an indication of a successful azetidinium modification.

### Linear and Nonlinear Viscoelasticity of Pristine CNC Dispersions

**Figure 3:**
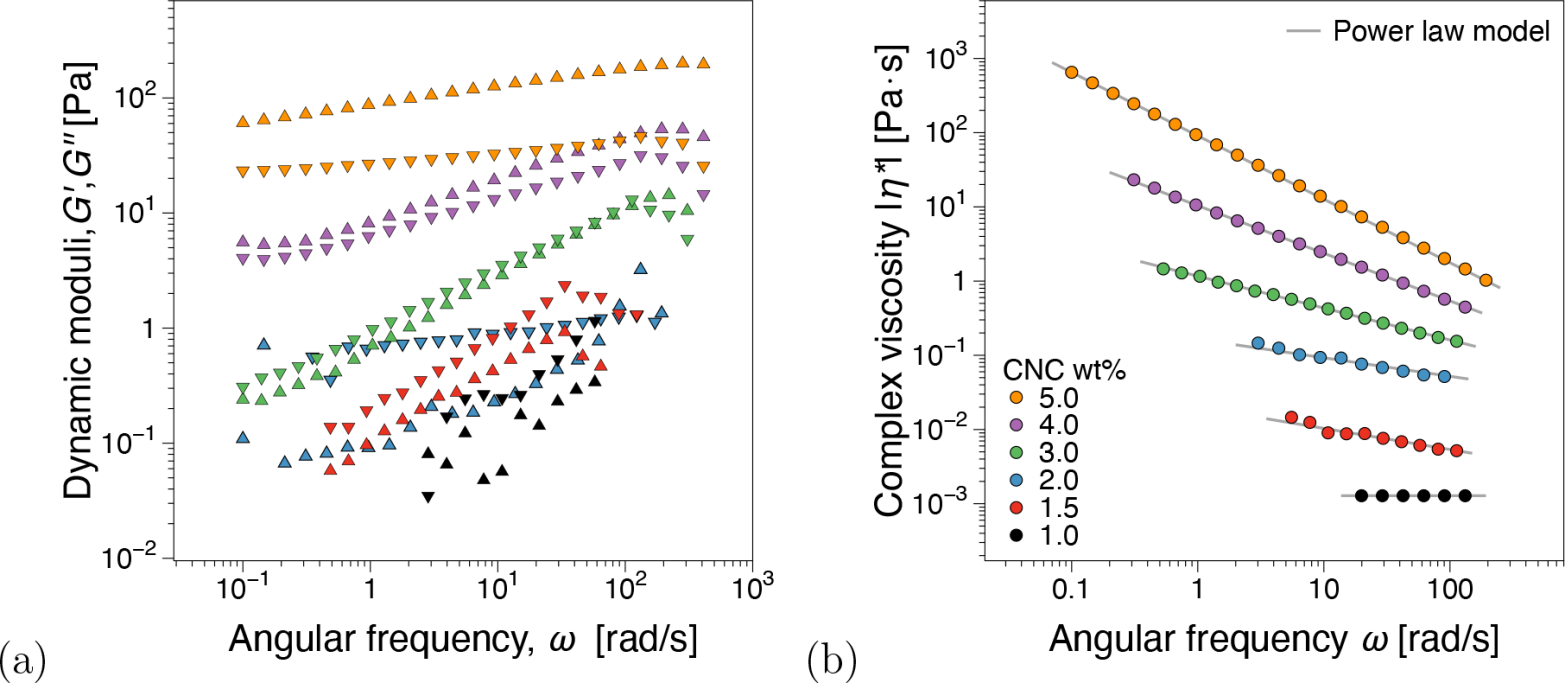
(a) Dynamic moduli, storage and loss moduli *G*′, *G*″(ω), and (b) complex viscosity functions, |η*|(ω), from linear viscoelastic frequency sweep measurements for pristine CNCs at different concentrations. Power law fit parameters can be found in Figure SI4 (Supporting Information).

From oscillatory shear frequency sweep tests, Figure 3a, concentrations below 2 wt % show a predominantly viscous dominated material response (*G*″ > *G*′). The gel point^42,43^ appears to be in the 3–4 wt % CNC range where *G*′ ≈ *G*″ independently of ω. An elastic-dominated response (*G*′ > *G*″) was recorded for 5 wt %. Although not emphasized in the discussion for brevity, the power law model, η = *K* · γ̇^*n*–1^, where *K* is the consistency and *n* is the flow index, fits of the viscosity functions in Figure 3b can be found in Figure SI5 (Supporting Information). In terms of *K* magnitude and *n* variation with increasing concentration, the fit parameters support the gel point as identified from dynamic moduli.

**Figure 4:**
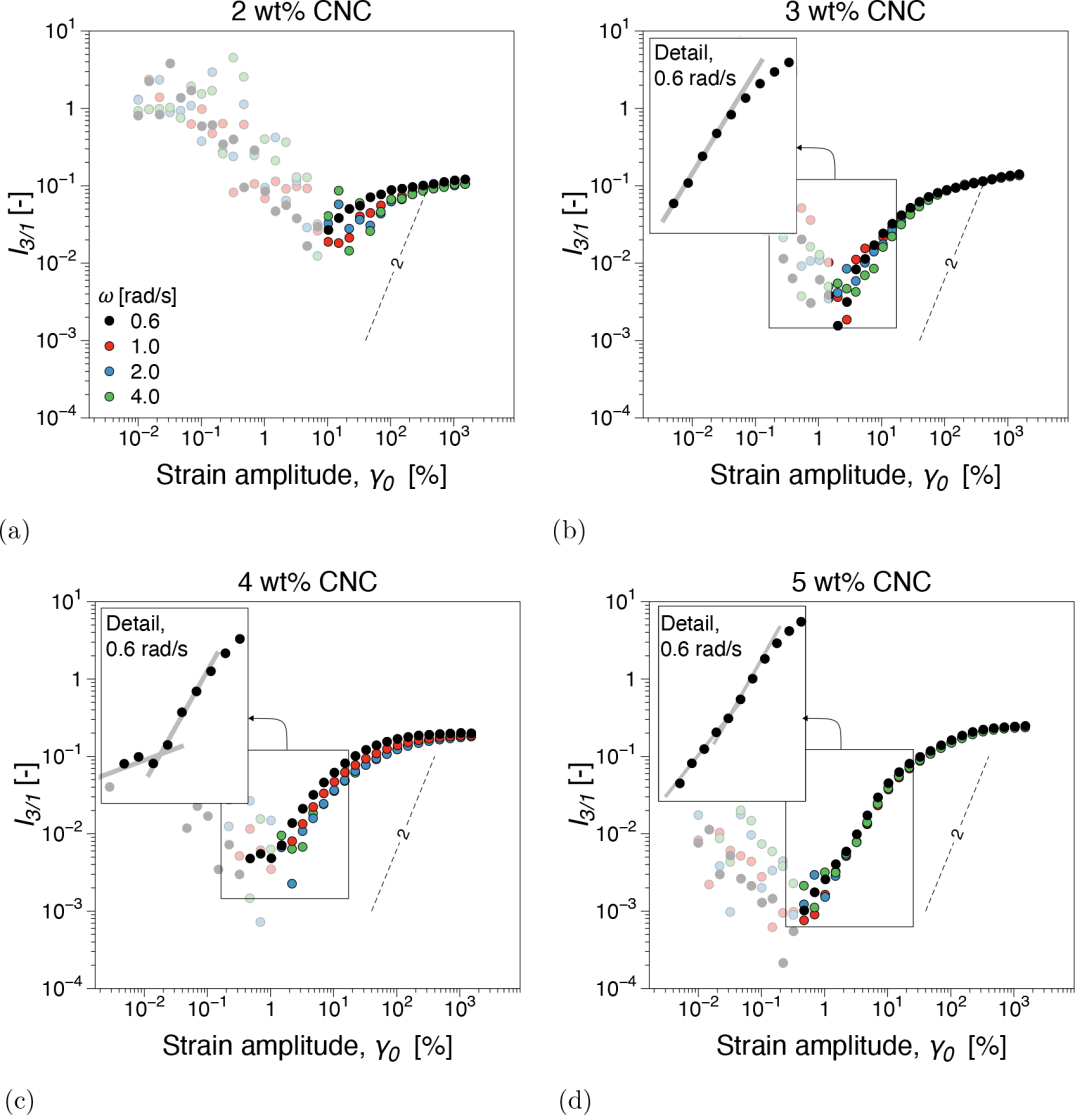
Third relative higher harmonic, *I*_3/1_, from dynamic strain sweeps for different concentrations of CNC–OSO_3_H suspensions: (a) 2, (b) 3, (c) 4, and (d) 5 wt %. The SAOS (small-amplitude oscillatory shear) region is characterized by instrumentation noise and is plotted as semitransparent noise.

**Figure 5:**
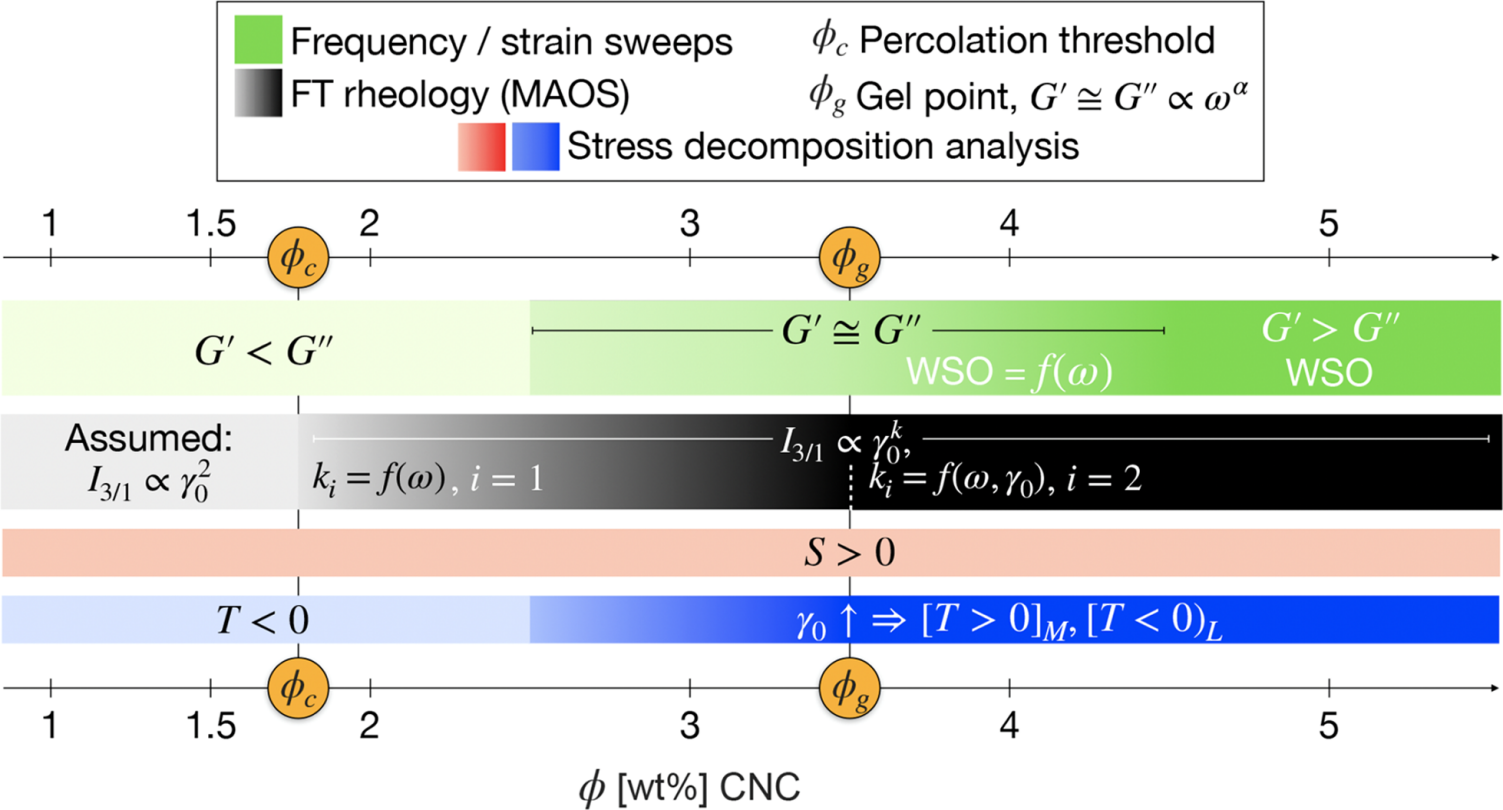
Comparative summary of linear and nonlinear viscoelastic parameters considered in the study highlighting their significance with increasing concentration for pristine CNC. For FT rheology, index ●_*i*_ refers to the number of discrete scaling regions distinguishable in the MAOS region.

The linear viscoelastic storage and loss modulus (*G*′, *G*″) of CNC-OSO_3_H suspensions from dynamic strain sweep measurements that form the basis for nonlinear analysis can be found in Figure SI4. The corresponding third-relative higher harmonic from FT-rheology, *I*_3/1_, and strain-stiffening and shear-thickening ratios, *S*, *T*, from stress decomposition can be found in Figure 4 and Figures SI6–SI8, respectively. Furthermore, a comparative visual summary of the linear and nonlinear parameters compared can be found in Figure 5, which we shall refer to throughout the discussion. For the lowest concentration investigated, 1 wt %, due to low measurement torques, the *ω* dependence of the dynamic moduli is likely an artifact, also considering the data in Figure 3. Consequently, their nonlinear material response can be largely attributed to noise. Despite more stable *G*′, *G*″ data, 1.5 wt % displayed nonlinear signatures rather challenging to interpret. Therefore, nonlinear data for 1 and 1.5 wt % can be found in Figure SI6. With respect to FT rheology, we refer to SAOS (small-amplitude oscillatory shear) as the *γ*_0_ range where *I*_3/1_ is dominated by experimental noise, typically as *I*_3/1_ ∝ γ_0_^*k*^, where *k* = −1.^44^ The MAOS region (medium-amplitude oscillatory shear) is in the strain amplitude range immediately after SAOS and has been theoretically predicted by a quadratic scaling law, *k* = 2. Any deviations from this theoretical scaling in the form of angular frequency and/or strain amplitude-dependent scaling regions are what we have dubbed nonlinear “oddities”.^45^ We refer to LAOS (large-amplitude oscillatory shear) as the strain amplitude region that follows.^29^ We briefly note that in some publications, the entire nonlinear analysis method is referred to as LAOS. However, most of our analysis is confined to the MAOS region as the LAOS region can be prone to experimental artifacts such as free surface distortions and slip.^46^ In particular, for *I*_3/1_, it is expected that both 1 and 1.5 wt % as isotropic suspensions would obey the theoretically predicted quadratic scaling region, i.e., *I*_3/1_ ∝ γ_0_^*k*^, where *k* = 2.^23^

At 2 wt % CNC, dynamic moduli appear reasonably stable and are convincingly viscous dominated when considering both frequency and strain sweep tests. In contrast, *I*_3/1_, Figure 4a, shows a nonlinear behavior that is *ω*-dependent and does not obey quadratic scaling in the MAOS region, whereas the data overlaps into the LAOS region. The angular frequency dependence comprises both the magnitude of *I*_3/1_ and the scaling in the MAOS region. The nonquadratic scaling behavior and angular frequency dependence of *I*_3/1_, i.e., nonlinear oddities,^47,48^ have been associated with the percolation threshold and subsequent network buildup in polymer nanocomposites containing electrically conductive fillers as well as other systems.^49^ In addition, we have previously shown similar results for (sulfated) commercial CNC suspensions and their desulfated counterparts.^23,32^ We, therefore, consider the nonlinear material signature described as evidence of a percolation threshold, ϕ_*c*_ in Figure 5, meaning that a weakly percolated network is formed that can be easily distorted by flow. With respect to stress decomposition, we focus on two nonlinear parameters, *S*, *T* i.e., the strain-stiffening and shear-thickening ratios. The two parameters are defined based on two elastic moduli and two dynamic viscosities based on the raw cycle data at minimum strain/rate, ●_M_, and large strain/rate, ●_L_: *S* ≡ (*G*_L_^′^ – *G*_M_^′^)/*G*_L_^′^ and *T* ≡ (*η*_L_^′^ – *η*_M_^′^)/*η*_L_^′^. Examples of raw cycle data in the form of elastic and viscous Lissajous–Bowditch diagrams can be found in Figure SI9, while Figure SI10 contains examples of the minimum and large strain/rate parameters.

In the 3–4 wt % concentration range, similar to the data in Figure SI4, *G*′ ≅ *G*″, supporting that the gel point, ϕ_g_, can be found within this range, see also Figure 5. Conversely, the *I*_3/1_ angular frequency dependence in the MAOS region, Figure 4b and 4c, is less pronounced with increasing concentration. A reduction in ω dependence as the CNC concentration increases toward the gel point, *ϕ*_c_ < ϕ < ϕ_g_, has been previously reported.^23,32^ Considering that the *I*_3/1_ scaling behavior at 3 wt %, Figure 4b is merely angular frequency dependent, *k* = *k*(ω), while at 4 wt %, Figure 4c, *k* = *k*(ω, γ_0_) at ω = 0.6 rad/s could be further evidence that ϕ_g_ ∈ 3, 4 wt % range. Concomitantly, in the same concentration range, the shear-thickening parameter, *T*, Figures SI7 and SI8b,c, can be inferred to exhibit a very weak at first (3 wt %) local intracycle nonlinear shear thickening, *T* > 0, approximately in the MAOS range, before transitioning to intracycle nonlinear shear thinning, *T* < 0.

In comparison, evidence of a weak strain overshoot (WSO), a local increase in *G*″ before transitioning to the nonlinear regime,^29^ can be seen for 4 wt % CNC in the dynamic moduli, Figure SI4a. However, the material structure responsible for jamming before nonlinear yielding appeared disrupted by ω, with WSO absent in Figure SI4b–d. We briefly note that the local shear-thickening behavior in *T* roughly corresponds to the WSO in *G*″. However, *T* appears to be, at least in certain cases, a more sensitive parameter to jamming/yielding in strain sweep.^32^ A WSO behavior independent of the applied ω was detected for 5 wt % CNC, Figure SI4. The consolidated network exhibited a rather weak γ_0_-dependent *I*_3/1_, Figure 4d, while the local shear-thickening behavior, *T* > 0, Figures SI7 and SI8d, was more pronounced.

Overall, network buildup and consolidation in pristine CNC suspensions having isotropic–isotropic gel transitions (non-self-assembling) bear both similarities and differences to our previous results on self-assembling CNC suspensions. First, the *k* = *k*(ω), where *I*_3/1_ ∝ γ_0_^*k*^, nonlinear scaling at percolation (*ϕ*_g_) is not only similar to other CNC suspensions^32^ but also similar when using different preparation methods,^28^ desulfated CNC systems,^23^ and even in nanocomposites.^45^ We can therefore emphasize that there is by now ample evidence supporting a scenario where a form of weakly interconnected networks is disrupted by the nonlinear deformations as the most striking nonlinearities (oddities) are recorded for the lowest imposed ω while with increasing ω there is a tendency toward the theoretically predicted quadratic MAOS scaling, *k* = 2. We note that in the self-assembling systems we have previously studied, both at percolation as well as at higher concentrations, the nonlinear signatures reported differ quite significantly from the ones reported in this study, especially in terms of the magnitude of the characteristic MAOS nonlinearity variation with ω, possibly suggesting a more agglomerated network at percolation with few connection points. Interestingly, a potentially unique insight is that the *I*_3/1_ scaling behavior around the gel concentration could suggest that, at least for isotropic gels, the gel point may not exhibit any nonlinear oddities.

### Linear and Nonlinear Viscoelasticity of Surface-Modified CNC-OSO_3_H Dispersions

In this section, the rheological properties of the modified suspensions are discussed for 1.5 and 3 wt %. Depending on the substituent and number of carbon atoms in the chain, networks formed by interparticle interactions of varying strength and mobility are expected to be created. Thus, we expect that this would be reflected in the network connectivity and rigidity and be readily observable as “fingerprints” from nonlinear oscillatory shear analysis. We note that surface modification may also be accompanied by a change in the dispersion state, which is inevitably a factor influencing rheological behavior.^23^ We divide the surface modifications into (i) the influence of branching, i.e., variation in a number of methine groups in the alkyl chains, and (ii) the influence of branch-on-branch structures, i.e., comparing linear alkyl groups with a 2-ethylhexyl group.

#### Influence of Branching

**Figure 6:**
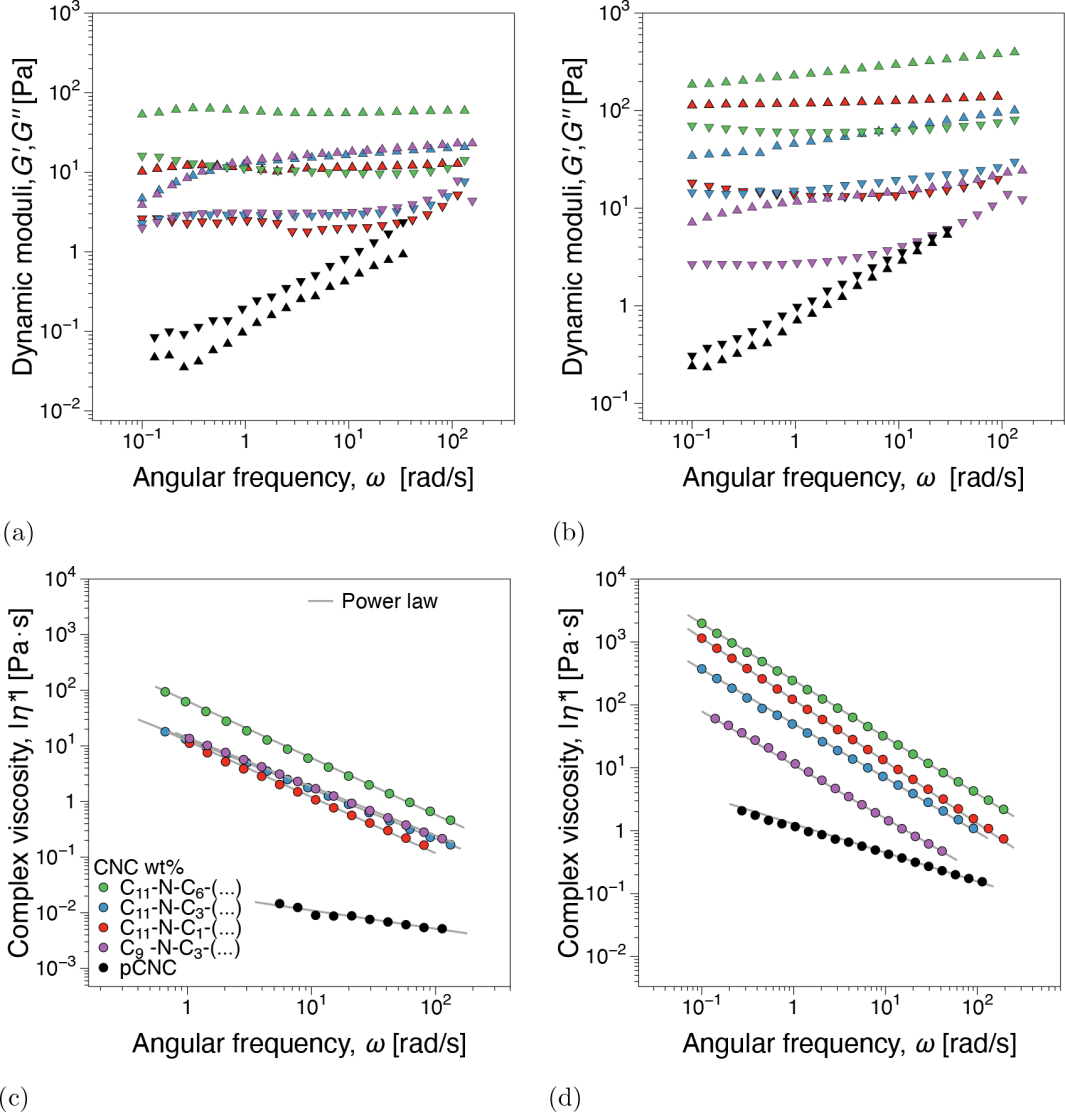
Dynamic storage and loss moduli, *G*′ and *G*″(ω), for (a) 1.5 and (b) 3 wt % and complex viscosity functions for (c) 1.5 and (d) 3 wt % from linear viscoelastic frequency sweep measurements.

The viscoelastic dynamic moduli and complex viscosity from frequency sweeps are presented in Figure 6, comparing the influence of branching and concentration in relation to the pristine CNC (pCNC). Power law fit parameters can be found in Figure SI16. Surface modification with azetidinium salts significantly impacts the viscosity and dynamic moduli. This is particularly significant for 1.5 wt %, where the pCNC is below the gel point, whereas all modified suspensions are rheological (isotropic) gels, *G*′ > *G*″, see Figure 6a and 6b. Longer linkers generally have a higher impact on the linear viscoelastic properties of the suspensions, whether branched or linear. Both linkers containing 12 carbons (C_11_-N-C_1_, C_9_-N-C_3_) exhibited lower complex viscosity compared to the linker with the highest total number of carbons (C_11_-N-C_6_). We interpret this linear viscoelastic behavior as the first evidence that the presence of the implemented surface substituents has structurally modified the CNC network.

**Figure 7:**
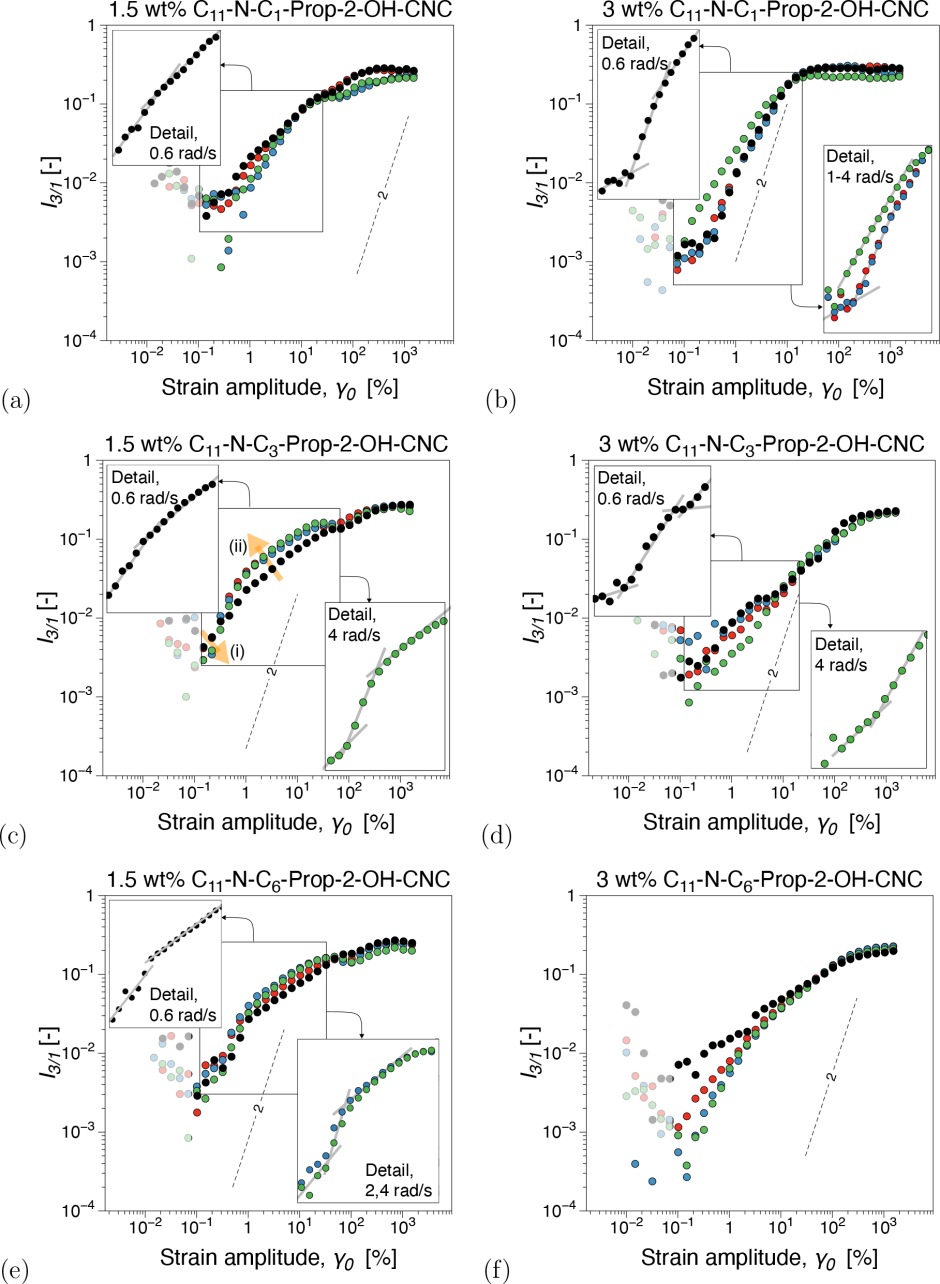
Third relative higher harmonic, *I*_3/1_, from dynamic strain sweep tests for branched C_11_-N-C*_m_*-Prop-2-OH-CNC, *m* = 1, 3, 6, substituents: (a) *m* = 1, 1.5 wt %, (b) *m* = 1, 3 wt %, (c) *m* = 3, 1.5 wt %, (d) *m* = 3, 3 wt %, (e) *m* = 6, 1.5 wt %, and (f) *m* = 6, 3 wt %. The SAOS (small-amplitude oscillatory shear) region is characterized by instrumentation noise and is plotted as semitransparent noise.

However, we wish to examine the central question of how the suspensions’ nonlinear microstructural dynamics, as identified through the nonlinear signatures analyzed, compared to the network buildup in pCNC. Chain mobility is expected to decrease with the number of carbons in the second branch, while the interaction strength is expected to increase with the number of carbons in the second branch (i.e., more branched surface linker topologies). The linear viscoelastic moduli from strain sweep tests are shown in Figure SI11, whereas the corresponding *I*_3/1_ are shown in Figures 7 and SI12, and *S* and *T* from stress decomposition analysis are shown in Figures SI13 and SI14. A comparative summary of the surface-modified CNC suspensions from the perspective of all of the linear and nonlinear material parameters investigated in this study can be found in Figure 10.

Several differences compared to the pCNC are readily observable right from the dynamic moduli in strain sweep tests, Figure SI11. First, the linear viscoelastic range, i.e., where the dynamic moduli are independent of the strain amplitude, was significantly reduced compared to the respective pCNC concentrations. Second, in terms of magnitude (similar observation can be made of the frequency-dependent moduli in Figure 6), either the moduli are comparable, e.g., Figure SI11a,d,e, or there is a slight increase in moduli with increasing ω. This suggests that the applied mechanical excitation could induce a stiffening effect. This is further underscored by the occurrence of WSO (*G*″, linear–nonlinear transition, which is more pronounced for higher ω).

Considering the nonlinear signatures of the surface-modified CNC suspensions, it is clear that their microstructural dynamics are distinct from pCNC network structures. Referring to *I*_3/1_, all surface-modified suspensions displayed significant nonlinear oddities; however, they also appear distinct and more challenging to interpret compared to those recorded in pCNC suspensions. In contrast to pCNC, the nonlinear behavior contains both ω and γ_0_ dependence of the scaling factor *k*, where *I*_3/1_ ∝ γ_0_^*k*^. However, it should be noted that it is possible that there could be closer nonlinear similitude between the surface-treated CNC suspensions and concentrations of pCNCs higher than considered in this work.

Interestingly, there appears to be a clear distinction between nonlinear signatures with increasing branching and concentration, see Figure 10. With increasing branching, for 1.5 wt %, the scaling behavior in *I*_3/1_ suggests that with increasing imposed ω there is a tendency toward microstructural restructuring generally in two stages (see also the orange arrows in Figure 7c). (i) In the low-γ_0_ range of the MAOS region, there is a sometimes lowering of the scaling exponents *k* with increasing ω slightly toward *k* → 2, see, e.g., Figure 7a for ω = 2, 4 rad/s. This can also be observed in Figure 10 (bubble chart *k* vs ω as the low*I*_3/1_ points plotted proportionally to the magnitude of the nonlinearity of the shear stress response, increase with ω toward *k* = 2, especially for the more branched surface linkers. (ii) In the higher γ_0_ range, toward the transition to the LAOS region, there is generally an increase in *I*_3/1_ magnitude. This is visualized in Figure 10 as larger circles, as the corresponding magnitude in *I*_3/1_ is higher. Interestingly, while small differences can be observed in magnitude, the scaling factor remains practically unchanged for the two nonlinear surface topologies, in contrast to the linear surface linker. This can also be inferred from the stress decomposition analysis, particularly in the shear-thickening ratio, *T*, see Figures SI13 and SI14. Thus, for C_11_-N-C_1_, Figures SI13 and SI14a, there appears to be a dual weak overshoot in *T* (local intracycle shear-thickening, *T* > 0) before transitioning to intracycle nonlinear shear thinning with increasing γ_0_. Moreover, increasing branching results in one weak overshoot in *T* in the low range of nonlinear γ_0_ and a region of approximate constant intracycle shear-thinning *S* ≈ 0 (also represented Figure 10). The dual-component distortion in nonlinear data could suggest a nonlinear microstructural reorganization in flow that possibly refers to two distinct microstructural processes, i.e., flow-induced distortions. These could correspond to the topology of the percolated network, suggesting perhaps larger agglomerate clusters with few links in between^31^ and that nonlinear deformations distort the connection points and the more dense areas (aggregates) in different strain amplitude ranges. We note that such a network structure could also be inferred for pCNC suspensions. However, the described nonlinear material response is absent, suggesting that the nonlinear network response to large deformations is strongly mediated by the presence of linkers at the CNC–CNC interface.

In contrast, for 3 wt %, increasing the number of carbons in the secondary branch results in apparent microstructural distortions mostly confined to the lower range of γ_0_ in the MAOS region. This is likely because of the higher concentration, whereby the network is expected to be more interconnected/consolidated, with oscillation-induced microstructural distortions likely dominated by large CNC aggregate distortions. Again, the complexity of the nonlinear signatures increases with increasing both branching and branching degree, with a significant ω dependence of the nonlinear material response detected for. Furthermore, considering the flexible C_11_-N-C_1_ linkers at 3 wt %, it shows an opposite trend with increasing ω compared to 1.5 wt % (and the two branched counterparts as a matter of fact): nonlinear material scaling response in *I*_3/1_ diverges from *k* = 2 for comparable γ_0_ to 1.5 wt %. This could correspond to the flow-induced aggregate swelling behavior previously reported.^31^ However, as the mobility of the linker is impaired by the addition of branches and the interaction strength increases, such behavior is no longer observed for the branched topologies. This could suggest that more branched linkers likely create a more rigid interconnected CNC network. It is less prone to oscillatory flow-induced distortions with increasing angular frequency in nonlinear conditions. Interestingly, from stress decomposition, there is a considerable local shear-thickening region *T* > 0 for the linear, Figures SI13 and SI14b, compared to the branched alternatives. In addition to the aspects already discussed, we note that 3 wt % linkers with C_11_-N-C_*m*_, with *m* = 1, 3, and 6, also contain nonlinear scaling regions with *k* ≈ 0, behavior that has previously been hypothesized to correspond to more agglomerated percolated networks.^47^ When additionally considering the linker with a reduced number of carbons in the longest alkyl chain (C_9_-N-C_3_), Figures SI13 and SI14a,b, it appears that a small reduction in the linker size influences the nonlinear material response for both concentrations investigated; see the corresponding *I*_3/1_ scaling parameters as well as *S*, *T* in Figure 10. Similarly, Figures SI13 and SI14h disclose extensive evidence of a dual nonlinear material response in *T* with a weak intracycle shear-thickening behavior (*T* > 0) followed by a region of intracycle shear-thinning (*T* < 0).

#### Influence of Branch-on-Branch Structures

**Figure 8:**
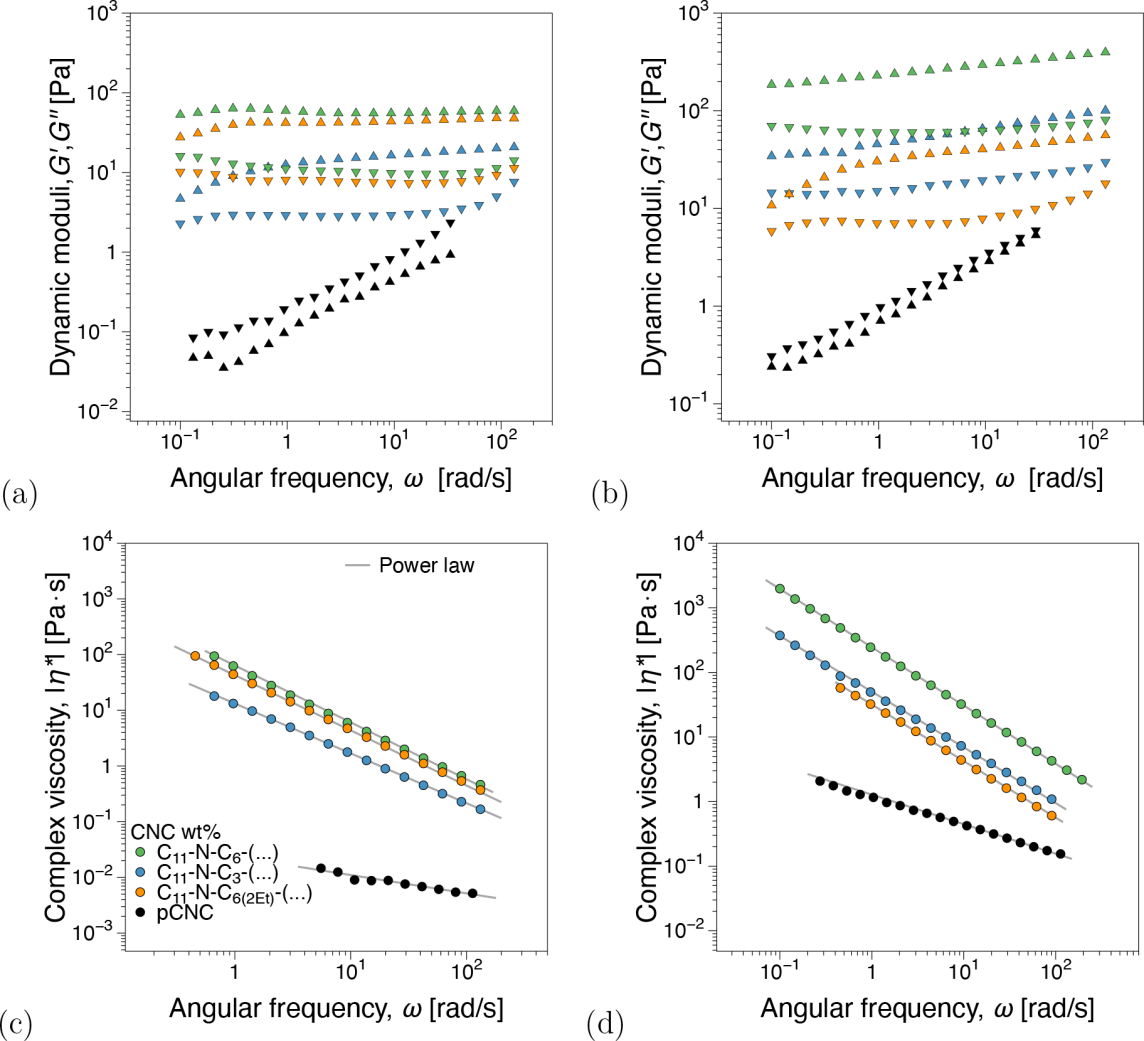
Dynamic storage (*G*′) and loss moduli (*G*″) for (a) 1.5 and (b) 3 wt % and complex viscosity functions, |η*|(ω, ϕ), for (c) 1.5 and (d) 3 wt % from linear viscoelastic frequency sweep measurements.

**Figure 9:**
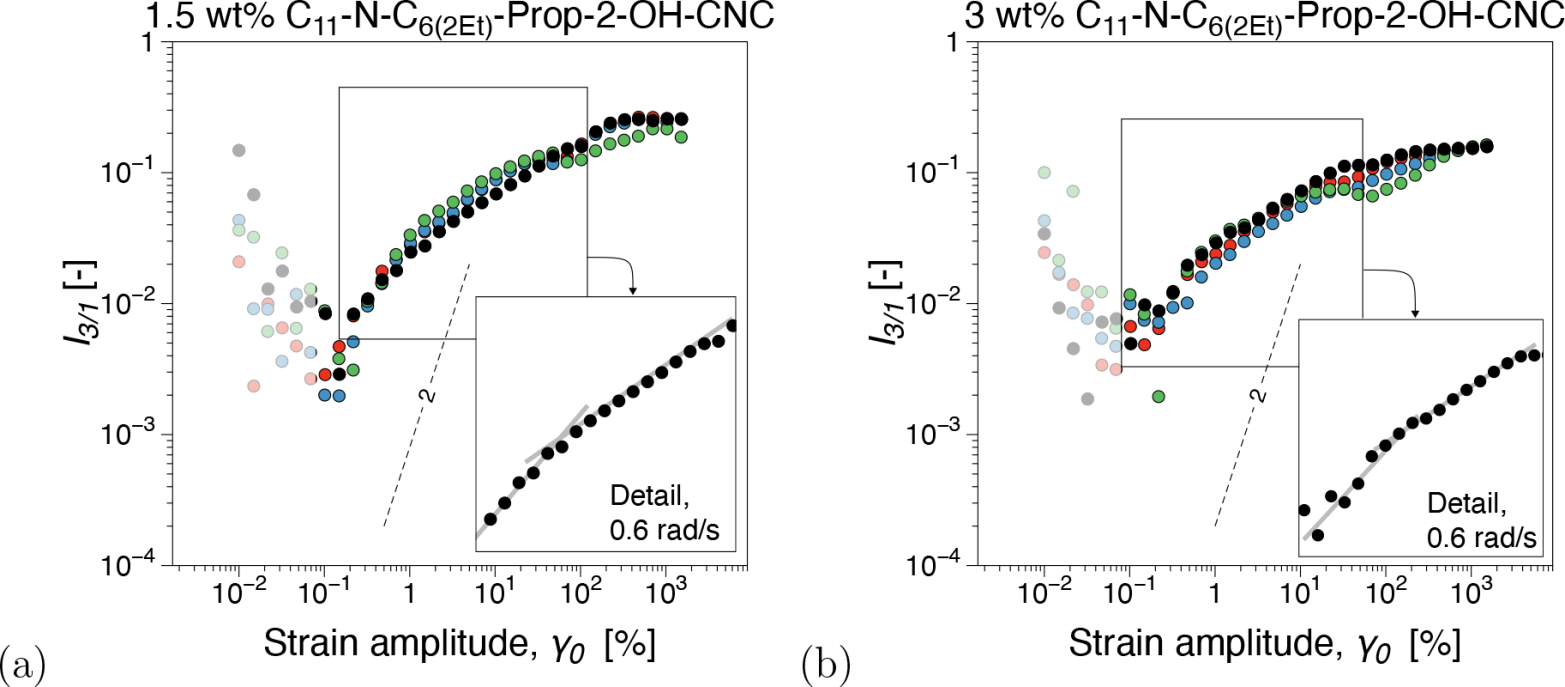
Third relative higher harmonic, *I*_3/1_, from strain sweep measurements for C_11_-N-C_6(2Et)_: (a) 1.5 and (b) 3 wt %. The SAOS (small-amplitude oscillatory shear) region is characterized by instrumentation noise and is plotted as semitransparent data.

To investigate whether the further reduction in grafted chain mobility and expected increase in interaction strength for more branched linkers plays a significant role, we also prepared one branch-on-branch linker C_11_-N-C_6(2Et)_. The linear viscoelastic data compared with the longer branched substituents (C_11_-N-C_3_, C_11_-N-C_6_), as they have chains equal to the fragments of branch-on-branch linkers, and pristine CNC are presented in Figure 8. Dynamic moduli from frequency sweeps show a similar trend, where C_11_-N-C_6(2Et)_ exhibits gel-like behavior, *G*′ > *G*″, Figure 8a and 8b. Figure 9shows the corresponding nonlinear material response as expressed by the third relative higher harmonic, *I*_3/1_.

**Figure 10:**
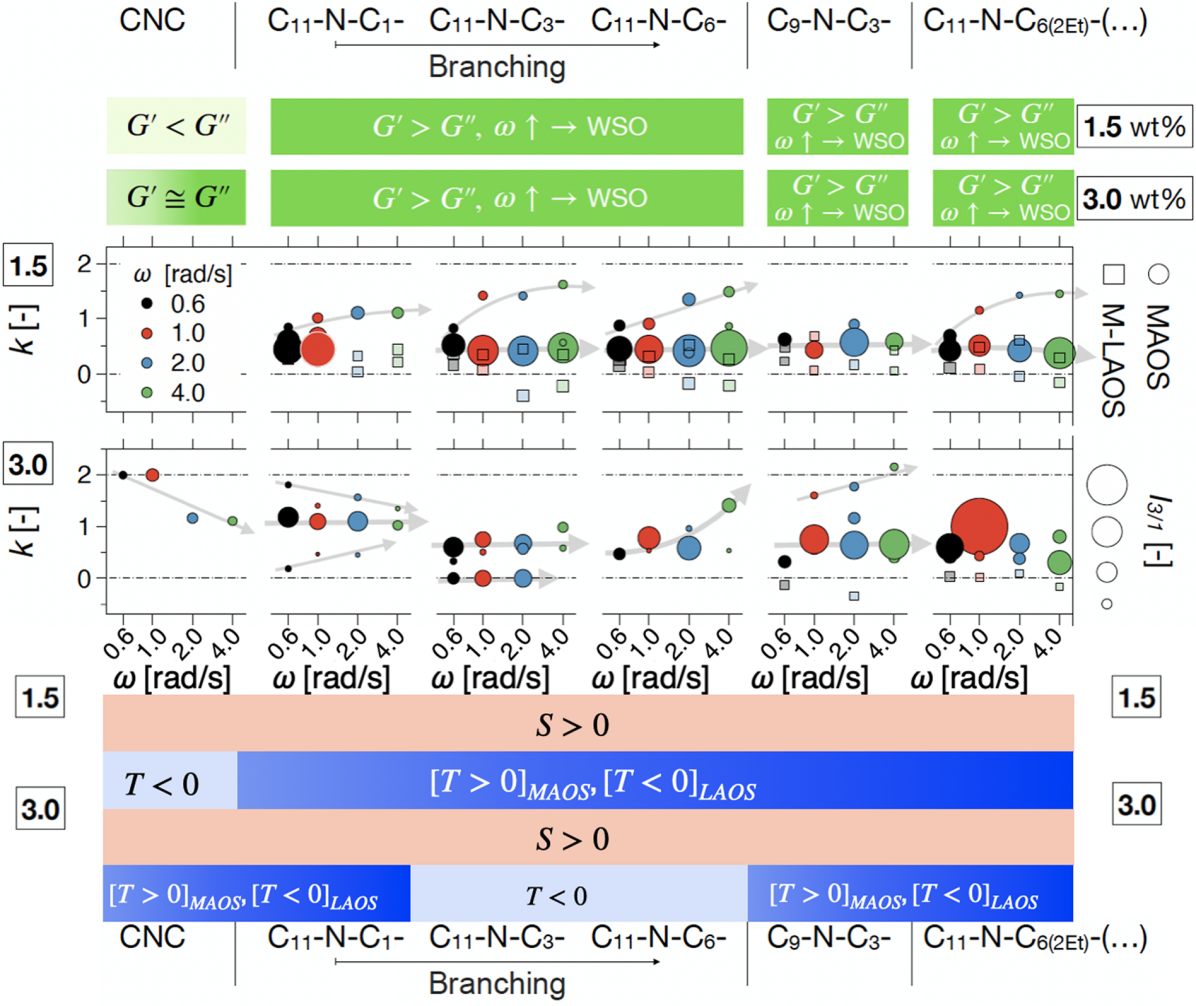
Comparative summary of linear and nonlinear viscoelastic parameters comparing pristine and surface-modified CNC suspensions. The circle plots relate simultaneously the MAOS scaling factors from FT rheology, *k*, in terms of how many there are and their magnitude, their ω dependence, as well as the magnitude of the nonlinearity as expressed by *I*_3/1_ for that particular scaling region through the size of the circles. Semitransparent squares refer to additional anomalous scaling factors at the MAOS–LAOS transition (M–LAOS in the legend on the right side).

The branch-on-branch structure showed dynamic moduli in strain sweep tests qualitatively and quantitatively comparable to other linkers, Figure SI11i,j. Interestingly, the complex viscosity of the branch-on-branch modifications is significantly lower than that for the other modified substituents. Similarly to the C_11_-N-C*_m_*-Prop-2-OH surface modifications, the dynamic moduli showed a linear viscoelastic threshold at low strain amplitudes and weak strain overshoot (WSO) at the higher angular frequencies investigated.

Considering the nonlinear signatures of the branch-on-branch topology compared to the other branched topologies, Figure 9, both *I*_3/1_ and *S*, *T* appear qualitatively similar to 1.5 wt % C_11_-N-C_*m*_-Prop-2-OH, *m* = 3 and 6, suspensions. This reinforces the conclusion that size could be a primary factor affecting the nonlinear material response with the branching degree being a secondary factor. However, for 3 wt %, the nonlinear material response in *I*_3/1_, albeit far from a quadratic scaling, does not show significant oddities, Figure 9b. At the same time, the elastic and viscous intracycle nonlinear material response is similar to C_9_-N-C_3_ with a local shear-thickening region followed by a constant shear-thinning region before LAOS, see, e.g., Figure SI15. This could be an indication that the reduction in linker mobility does have an impact at higher concentrations where a dense, more rigid, percolated network is likely present (3 wt %).

Although not part of the MAOS region and therefore likely prone to experimental errors, we note that at the MAOS–LAOS transitions, see the semitransparent square points in Figure 9, *I*_3/1_ shows significant unexpected scaling features, especially in all modified 1.5 wt % and less mobile short-branched C_9_-N-C_3_ and branch-on-branch C_11_-N-C_6(2Et)_.

## Conclusions

Tailored attractive (non-self-assembling) aqueous CNC suspensions were successfully prepared through the grafting of dialkyl groups on the surface of the CNCs, as confirmed through NMR, FTIR, and TGA analyses. As expected, the surface modifications had a significant impact on the linear viscoelastic material parameters in comparison to pCNC suspensions. However, the study focused on nonlinear material parameters from Fourier-transform rheology and stress decomposition analysis, as more sensitive alternatives for rheological characterization. Nonlinear material parameters, in particular, so-called 'oddities' in the form of angular frequency and/or strain amplitude-dependent non-quadratic scaling in the third relative higher harmonic, *I*_3/1_, appear to detect a percolation threshold before it could be inferred from linear viscoelastic data. Regardless of the materials response of the pCNC suspensions, the surface-modified CNC suspensions exhibited an elastic-dominated material response at both concentrations investigated, 1.5 and 3 wt %, i.e., were isotropic gels. Nonlinear material parameters were interpreted in terms of network connectivity (concentration and surface linker topology) as well as varying in surface chain mobility, varying from linear to branch-on-branch topologies, and strength of interaction, increasing from branched to linear topologies. It was found that linear vs branched and the length of the grafted chains were the primary factors influencing the nonlinear material response of the isotropic gels. Furthermore, less interconnected networks (1.5 wt %) showed evidence of two distinct processes. These likely correspond to connection points between aggregates and the more dense areas (aggregates) of the network being distorted by the nonlinear deformations in different strain amplitude ranges. Overall, the nonlinear rheological behavior of aqueous CNC systems and surface-modified isotropic gels is nontrivial and could provide insight into a series of physical processes occurring as a function of concentration and applied deformation/deformation rates. However, their interpretation is, for now, limited to conjectures related to particle–particle interactions and network and aggregate distortions.

## Supporting Information Available

Experimental details; typical potentiometric titration curves for sulfated cellulose nanocrystals (CNC) against NaOH; FTIR spectra; chemical structures and nomenclature of dialkyl amine and dialkyl azetidinium molecules; dynamic storage (*G*′) and loss moduli (*G*′′) for all concentrations of CNCOSO_3_H from strain sweep tests; power law model parameters, the consistency index *K* and the flow index *n*, from fittings of the complex viscosity functions in Figure 2a; nonlinear material response represented by the third relative higher harmonic, *I*_3/1_, from dynamic strain sweeps for different concentrations of CNCOSO_3_H suspensions; *S*, *T* parameters for pristine CNC; scalar plots of strain-stiffening, *S*, and shear-thickening, *T*, nonlinear material parameters for ω = 0.6, 1, 2, 4 rad/s; Lissajous–Bowditch (LB) diagrams for CNCOSO_3_H suspensions; minimum strain rate *G*′ _L_ and large strain rate *G*′ _M_ moduli of nonlinear material parameters; dynamic storage (*G*′) and loss moduli (*G*′′) for branched C_11_-N-C_*m*_-Prop-2-OH-CNC, C_9_-N-C_3_-Prop-2-OH-CNC, and C_11_-N-C_6(2Et)_-Prop-2-OH-CNC strain sweep measurements; nonlinear material response represented by the third relative higher harmonic, *I*_3/1_, from dynamic strain sweeps for different concentrations of C_9_-N-C_3_-Prop-2-OH-CNC suspensions from strain sweep measurements; *S*, *T* parameters for branched C_11_-N-C_*m*_-Prop-2-OH-CNC and C_9_-N-C_3_-Prop-2-OH-CNC from strain sweep measurements; *S*, *T* parameters for branched C_11_-N-C_*m*_-Prop-2-OH-CNC from strain sweep measurements; *S*, *T* parameters for branched-on-branch structure; power law model parameters from fittings of the complex viscosity functions in Figure 7; loss tangent, tan δ = *G*′′/*G*′, from linear viscoelastic strain sweep measurements as a function of branched linkers for both investigated concentrations for all applied angular frequencies; power law model parameters, the consistency index *K*, and the flow index *n* from fittings of the complex viscosity functions; summary of CNC chemical characterization data (PDF)
