# Supplementary material for: Isotropic Gels of Cellulose Nanocrystals Grafted with Dialkyl Groups: Influence of Surface Group Topology from Nonlinear Oscillatory Shear
Source: Langmuir. 2023 Apr 25;39(18):6433–46. doi: 10.1021/acs.langmuir.3c00210 (PMC10173451; doi:10.1021/acs.langmuir.3c00210)
Supplement: Supplementary file 1 — la3c00210_si_001.pdf [file la3c00210_si_001.pdf]

Isotropic gels of cellulose nanocrystals grafted with dialkyl groups: influence of surface group topology from nonlinear oscillatory shear

Sylwia Wojno,[†,¶] Amit Kumar Sonker,[‡,¶] Jelka Feldhusen,[‡] Gunnar Westman,[*,‡,¶] and Roland Kádár[*,†,§]

†*Department of Industrial and Materials Science, Division of Engineering Materials, Chalmers University of Technology, SE-412 96 Gothenburg, Sweden*

‡*Department of Chemistry and Chemical Engineering, Division of Chemistry and Biochemistry, Chalmers University of Technology, SE-412 96 Gothenburg, Sweden*

¶*Wallenberg Wood Science Center (WWSC), Chalmers, SE-412 96 Gothenburg, Sweden*

§*Wallenberg Wood Science Center, Chalmers, SE-412 96 Gothenburg, Sweden*

E-mail: westman@chalmers.se; roland.kadar@chalmers.se

List of Figures

1 Typical potentiometric titration curves for sulfated cellulose nanocrystals (CNC) against NaOH. 5

2 (a) FTIR spectra from 855-770 $cm^{-}1$ indicate shift for C-O-S for modified CNC samples from 814 to 808 $cm^{-}1$ in comparison to pristine CNC samples, (b) FTIR Spectra from 4000 to 2500 cm-1 particularly, first derivative of the alkyl band. The short, dashed lines, I, II, III and IV corresponds to –CH3 (anti symmetric stretch at 2970-2950 cm-1), CH2 (anti symmetric stretch at 2940-2915 cm-1), -CH2 (symmetric stretch at 2870-2840 cm-1) and –CH3 (symmetric stretch at 2885-2860 cm-1), respectively. 7

3 Chemical structures and nomenclature of dialkyl amine and dialkyl azetidinium molecules: (a) dialkylamines, (b) dialkylamine azetidinium salts (open), (c) dialkylamine azetidinium salts (closed). 12

4 Dynamic storage (G') and Loss moduli (G'') for all concentrations of CNC-OSO_3H from strain sweep tests for $\omega =$ (a) 0.6, (b) 1, (c) 2 and (d) 4 rad/s. 13

5 Power law model parameters, the consistency index K and the flow index n, from fittings of the complex viscosity functions in Fig. 2 (a). 14

6 The nonlinear material response represented by the third relative higher harmonic, $I_{3/1}$, from dynamic strain sweeps for different concentrations of CNC-OSO_3H suspensions (a) 1, (b) 1.5 wt% and the stress decomposition parameters, strain-stiffening, S, and shear-thickening, T (c) 1, (d) 1.5 wt%. 15

7 S, T parameters for pristine CNC: (a) 2 wt%, (b) 3 wt%, (c) 4 wt%, (d) 5 wt% from strain sweep measurements. 16

8 Scalar plots of strain-stiffening, S, and shear-thickening, T, nonlinear material parameters for $\omega = 0.6, 1, 2, 4$ rad/s: (a) 2, (b) 3, (c) 4, (d) 5 wt%. The color intensity represents the S, T magnitudes and sign according to the color ramps. To help identifying especially changes in sign and the transition to nonlinear material response, iso-S, T Contours lines for $S, T = -1, 0, 1$ are overlaid as dotted lines and '+' and '-' sign is indicate a change in sign in the nonlinear region. 17

9 Lissajous-Bowditch (LB) diagrams for CNC-OSO_3H suspensions for selected $\omega = 0.6, 1, 4$ rad/s for (a,d) 1 wt%, (b,e) 1.5 wt%, (c,f) 5 wt% compiled as Pipkin diagrams. Top row, (a-c) represent the elastic LB diagrams and bottom row, (d-f) represent the viscous LB diagrams. 18
10 Minimum strain rate G'_L and large strain rate G'_M moduli, nonlinear material parameters for $\omega = 0.6, 1, 2, 4$ rad/s: (a) 1 wt%, (b) 1.5 wt%, (c) 2 wt%, (d) 3 wt%, (e) 4 wt%, (f) 5 wt%. 19
11 (continued) 20
11 Dynamic Storage (G') and Loss moduli (G'') for branched: 11-N-m-Prop-2-OH-CNC, $m = 1, 3, 6$, substituents: (a) $m = 1$ 1.5 wt%, (b) $m = 1$ 3 wt%, (c) $m = 3$ 1.5 wt%, (d) $m = 3$ 3 wt%, and (e) $m = 6$ 1.5 wt% (f) $m = 6$ 3 wt%, 9-N-3-Prop-2-OH- (g) 1.5 wt%, (h) 3 wt% and 11-N-6(2Et)-Prop-2-OH-CNC (i) 1.5wt% and (j) 3 wt% from strain sweep measurements. 21
12 The nonlinear material response represented by the third relative higher harmonic, $I_{3/1}$, from dynamic strain sweeps for different concentrations of 9-N-3-Prop-2-OH-CNC suspensions a) 1.5, b) 3 wt% from strain sweep measurements. 22
13 (continued) 23
13 S, T parameters for branched: C_{11}-N-m-Prop-2-OH-CNC, $m = 1, 3, 6$, substituents: (a) $m = 1$ 1.5 wt%, (b) $m = 1$ 3 wt%, (c) $m = 3$ 1.5 wt%, (d) $m = 3$ 3 wt%, (e) $m = 6$ 1.5 wt% (f) $m = 6$ 3 wt%, and C_9-N-C_3-Prop-2-OH-CNC (g) 1.5 wt%, (h) 3 wt% from strain sweep measurements. 24
14 S, T parameters for branched: C_{11}-N-m-Prop-2-OH-CNC, $m = 1, 3, 6$, substituents: (a) $m = 1$ 1.5 wt%, (b) $m = 1$ 3 wt%, (c) $m = 3$ 1.5 wt%, (d) $m = 3$ 3 wt%, (e) $m = 6$ 1.5 wt% (f) $m = 6$ 3 wt% from strain sweep measurements. More details about the colormaps can be found in the caption of Fig. 8. . . . 25
15 S, T parameters for branched-on-branch structure: (a) 1.5 wt%, (b) 3 wt%, visualization as the colormaps: (c) 1.5 wt%, (d) 3 wt%. 26

16 Power law model parameters, (a) the consistency index K and (b) the flow index n, from fittings of the complex viscosity functions in Fig. 7. 27
17 Loss tangent, $\tan\delta = G''/G'$, from linear viscoelastic strain sweep measurements as function of branched linkers for both investigated concentrations for all applied angular frequencies. 27
18 Power law model parameters, the consistency index K and the flow index n, from fittings of the complex viscosity functions in Fig. 9, (a) 1.5 wt%, (b) 3 wt% modified CNCs. 28

List of Tables

1 Summary CNC chemical characterization data. 6

Supplementary information

Materials Microcrystalline Cellulose (MCC) PH-101, Sulfuric Acid (98%), Undecanal, Methylamine, Nonanal, Propylamine, Hexyl amine, 2-Ethyl hexyl amine, Epichlorohydrin, Magnesium Sulfate, Sodium Borohydride Ethanol, Tetrahydrofuran, Chloroform, and Isopropanol were purchased from Sigma Aldrich (now Merck).

1.2 Methods

Determination of sulfate content by potentiometric titration

The sulfate half ester content of each CNC sample was determined through a potentiometric titration. Samples of 0.5% CNC were prepared through dilution using Milli-Q water (ultra-pure water) and sonicated 5 times for 1 minute (40% amplitude, 20 kHz) on VC505 Vibra Cell (Sonics, Connecticut USA). The potentiometric titration is preferred over the conductometric titration for determining the surface charge of nanoparticles in water because it produces higher accuracy and precision.[1] The titration device Titrando 905 (Metrohm) equipped with a pH glass electrode, model Unitrode easy Clean (Pt100, pH range: 0-14;

temperature range: 0-100° C) was used in the titration experiments. Prior to titration, cellulose nanocrystals suspension was purged with N2 gas for 10 minutes. With the flow of nitrogen gas inside the glass vessel, an inert atmosphere and 25° C temperature were maintained throughout the experiment. By adding NaOH, the pH titration went from 3.4 to 10.1. Based on the maximum ERC level (endpoint recognition criteria), the equivalnece point was determined.

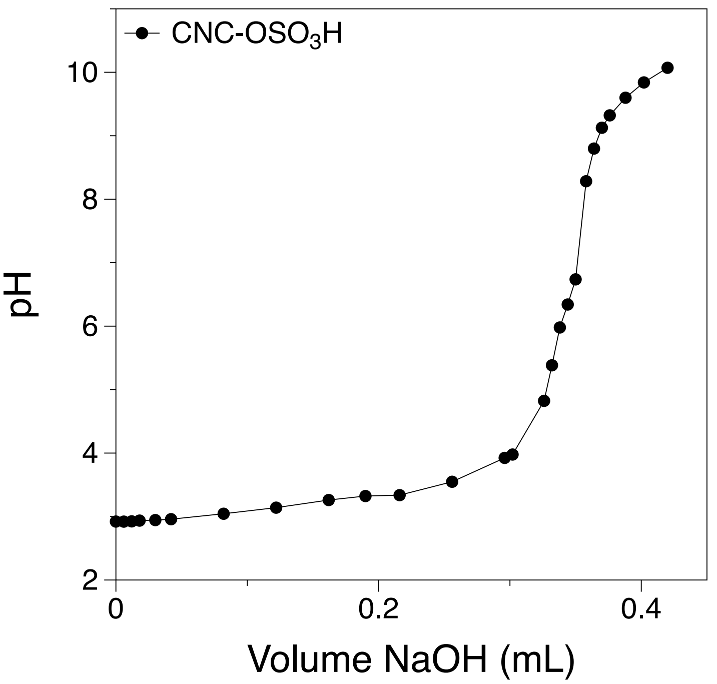

Figure SI 1: Typical potentiometric titration curves for sulfated cellulose nanocrystals (CNC) against NaOH.

The volume (V_eq) corresponds to equivalence point used to calculate the sulfate content by following equation:

$$Sulfatecontent(mmol/g) \equiv \frac{C \times V_{eq}}{M_s \times D_m} \times 100 \quad (1)$$

At the point of equivalence, C and V_{eq} are the concentration and volume of NaOH, while M_s is the total suspension weight and D_m is the suspension concentration.

Synthesis of Dialkylamines

Table 1: Summary CNC chemical characterization data.

Type of CNC	Degradation Onset Temperature [°C]	Residual char [wt%]	ζ-potential [mV]
pristine CNC	145.1	35.4	47.0
C_{11}-N-C_1-Prop-2-OH-CNC	231.2	24.2	31.0
C_{11}-N-C_3-Prop-2-OH-CNC	177.6	25.1	30.3
C_{11}-N-C_6-Prop-2-OH-CNC	218.6	21.3	32.1
C_9-N-C_3-Prop-2-OH-CNC	179.7	26.9	30.2
C_{11}-N-$C_{6(2Et)}$-Prop-2-OH-CNC	179.8	27.6	29.8

A solution containing of 10 mmol (1.7 g) of undecanal, 10 ml CH_3OH and 10 mmol (1.2 g) 1-ethyl-hexyl-amine were placed in a round bottom flask. Reductive amination was performed by stirring the mixture in ice bath for 1.5 h. Then, 0.3 mmol (0.1 g) $NaBH_4$ was added very slowly. The reaction was left for stirring at room temperature overnight. Next, rotor evaporator was used to evaporate the MeOH. The reaction mixture was extracted with CH_2Cl_2 and dried with anhydrous Na_2SO_4. Then, the crude product was isolated by rotor evaporation. The product was characterized by 1H and 13C NMR. Similar procedure was performed in the synthesis of all dialkylamines.

Synthesis of 1-chloro-2-hydroxy-propyl-N-dialkylamines

While stirring, to a solution containing 12 ml of isopropanol and 20 mmol of dialkylamine (1.94 g) was added dropwise 25 mmol of epichlorohydrin (2.31 g). The reaction was stirred overnight with a magnetic stirrer at room temperature. The solvent was rotary evaporated, and a sample of the crude product was characterized by 1H and 13C NMR as open form of azetidinium molecules. The crude product was dissolved in a mixture of H2O:IPA (9:1) whilst stirring. The reaction was stirred at room temperature for 30 min, and then the temperature was increased to 90° C for 2 h. The solvent was evaporated in a rotary evaporator and product was characterized by 1H and 13C NMR as closed form of azetidinium molecules.

The linkers were choosen/designed based on previous findings,[2] wherein it was found that C_{11}-N-C_1 linkers arranged in uniform structures during shear whereas linkers with C_6-N-C_6 did not form uniform structures. From molecular modelling it was realized the C_{11}-N_1 linker

has a more 'beehive'-like probability distribution whereas the C_6-N-C_6 linker had a more oblate like probability distribution. Thus, to gain a better understanding of how the surface structure affect the alignment and interaction between crystallites we made the following set of surface modification. The C_{11}-N-R, R=1, 3, 6 surface groups were chosen with the assumption that the longer the R group are, the less uniform the interaction would become. The second set, based on C_9-N-C_3 and $C_{6(2Et)}$ with the assumption that the extra branching would give an even more oblate distribution giving a larger hydrophobic areas on the surface. It shall be stressed that in the referred article (Wojno et. al[2]) the sulfate half ester content on the CNC surface was 170 μmol/g whereas in this study the surface charge is 330 μmol/g.

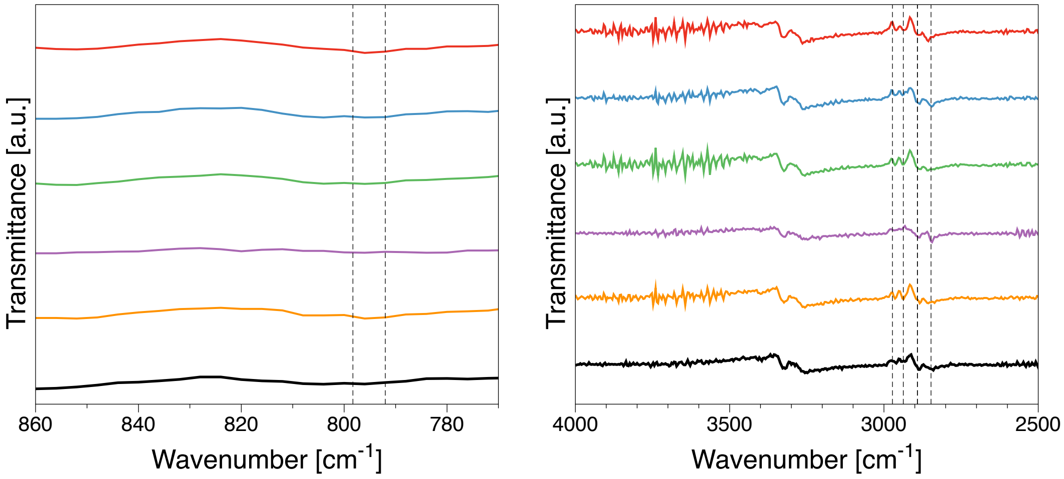

(a) (b)

Figure SI 2: (a) FTIR spectra from 855-770 cm^{-}1 indicate shift for C-O-S for modified CNC samples from 814 to 808 cm^{-}1 in comparison to pristine CNC samples, (b) FTIR Spectra from 4000 to 2500 cm-1 particularly, first derivative of the alkyl band. The short, dashed lines, I, II, III and IV corresponds to –CH3 (anti symmetric stretch at 2970-2950 cm-1), CH2 (anti symmetric stretch at 2940-2915 cm-1), -CH2 (symmetric stretch at 2870-2840 cm-1) and –CH3 (symmetric stretch at 2885-2860 cm-1), respectively.

NMR data for dialkylamines

- N-methylundecan-1-amine C_{11}-N-C_1

  ^1H NMR (400 MHz, CDCl3) δ 0.88, t, J= 7Hz (3H), 1.2-1.3, broad, (16H), 1.47, m, J=8Hz, 2.43, s, (3H), 2.55, t, J=7 Hz, (2H)

^{13}C NMR (101 MHz, CDCl3) δ 14.60, 23.16, 27.83, 29.81, 30.07, 30.08, 30.09, 30.10,30.42, 32.38, 37.05, 52.71

- N-propylundecan-1-amine C_{11}-N-C_3

  ^{1}H NMR (400 MHz, CDCl3) δ 0.88, t, J=8Hz, (3H),0.91, t, J=8Hz, (3H), 1.20-1.34, multiplet, (12H) 1.42-1.55, multiplet, (4H), 2.56, t, J=8Hz, (2H), 2.58, t, J=8Hz, (2H)

  ^{13}C NMR (101 MHz, CDCl3) δ 11.83, 14.11, 22.67, 23.27, 27.43, 29.28, 29.57, 29.60, 30.23, 31.88, 50.12, 52.04.

- N-hexylundecan-1-amine C_{11}-N-C_6

  ^{1}H NMR (400 MHz, CDCl3) δ 0.88, t, J=8Hz, (3H), 0.89, t, J=8Hz, (3H), 1.22-1.34, multiplet, (22H), 1.45-1.54, bm, (4H), 2.58, t, J=8Hz, (4H)

  ^{13}C NMR (101 MHz, CDCl3) δ 14.21, 14.27, 22.77, 22.83, 27.03, 27.25, 27.51, 27.82, 29.49, 29.73, 29.75, 29.79, 30.24, 31.95, 32.06, 50.25.

- N-propylnonan-1-amine C_9-N-C_3

  ^{1}H NMR (400 MHz, CDCl3) δ 0.78 – 0.95 (m, 6H), 1.13 – 1.41 (m, 12H), 1.49 (dt, J = 13.4, 6.8 Hz, 4H), 2.57 (dd, J = 15.4, 7.8 Hz, 4H)

  ^{13}C NMR (101 MHz, CDCl3) δ 11.78, 14.08, 22.65, 27.40, 29.26, 29.55, 31.87, 50.03, 51.94.

- N-(2-ethylhexyl) Undecan-1-amine $C_{6(2Et)}$-N-C_{11}

  ^{1}H NMR (400 MHz, CDCl3) δ 0.87, t, J=8Hz, (3H), 0.89, t, J=8Hz, (6H), 1.22-1.40, broad, (24 H), 1.45-1.55, broad, (3H), 2.52, d, J=8Hz, (2H), 2.61, J=8Hz, (2H)

  ^{13}C NMR (101 MHz, CDCl3) δ 10.60, 13.97, 13.98, 22.53, 22.94, 24.21, 27.20, 28.74, 29.18, 29.20, 29.38, 29.41, 29.44, 29.46, 31.12, 31.75, 38.77, 49.98, 52.84.

- NMR data for dialkylamine Azetidinium Salts molecules (Open form)

  1-chloro-3-(methyl(undecyl)amino) propan-2-ol [C_{11}-N-C_1-Prop-2-OH (O)]

^{1}H NMR (400 MHz, CDCl3) δ 0.88, t, 8=Hz, (3H) 1.20-1.32, broad singlet, (16H), 1.42-1.48, broad (2H), 2.26, s (3H), 2.32-.40, broad, (2H), 2.43-2.58, broad, (2H), 3.55, d, J=8Hz, (2H), 3.85, multiplet, (1H)

^{13}C NMR (101 MHz, CDCl3) δ 14.28, 22.83, 27.40, 27.43, 29.48, 29.70, 29.77, 32.06, 42.29, 47.28, 58.24, 60.71, 67.06.

- 1-chloro-3-(propyl(undecyl)amino) propan-2-ol [C_{11}-N-C_3-Prop-2-OH (O)]

  ^{1}H NMR (400 MHz, CDCl3) δ 0.88, t, 8=Hz, (6H), 1.26, broad singlet, (16H), 1.36-1.52, broad, (4H), 2.32-2.60, broad, (6H), 3.55, d, J=8Hz, (2H), 3.82, multiplet, (1H)

  ^{13}C NMR (101 MHz, CDCl3)δv 11.91, 14.28, 20.44, 22.84, 25.52, 27.27, 27.53, 29.49, 29.72, 29.77, 29,78, 32.06, 47.28, 54.49, 56.39, 57.80, 67.11.

- 1-chloro-3-(pentyl(undecyl)amino) propan-2-ol [C_{11}-N-C_6-Prop-2-OH (O)]

  ^{1}H NMR (400 MHz, CDCl3) δ 0.88, t, 8=Hz, (6H), 1.25, broad singlet, (22H), 1.35-1.55, broad, (4H), 2.36-2.65, 2.61,broad (6H), 3.55, d, J=8Hz, (2H), 3.81, multiplet, (1H).

  ^{13}C NMR (101 MHz, CDCl3) δ 14.21, 14.28, 22.79, 22.84, 27.19, 27.23, 27.26, 27.52, 29.49, 29.71, 29.77, 31.91, 32.06, 47.29, 54.48, 57.79, 67.10.

- 1-chloro-3-(nonyl(propyl)amino) propan-2-ol [C_9-N-C_3-Prop-2-OH (O)]

  ^{1}H NMR (400 MHz, CDCl3) δ 00.88, t, 8=Hz, (3H) 0.94, t, 8=Hz, (3H) 1.20-1.36, broad singlet, (12H), 1.37-1.57, multiplet, (4H), 2.35-2.55, broad, (4H), 2.55-2.65, dt, J=8Hz, (2H), 3.55, d, J=8Hz, (2H), 3.83, multiplet, (1H)

  ^{13}C NMR (101 MHz, CDC3) δ 11.91, 14.27, 20.44, 22.82, 27.26, 27.52, 29.43, 29.73, 32.02, 47.29, 51.94, 54.50, 56.39, 57.80, 67.11.

- 1-chloro-3-(pentyl(undecyl)amino) propan-2-ol [C_{11}-N-$C_6(2Et)$-Prop-2-OH (O)]

^1H NMR (400 MHz, cdcl3) δ 0.87, broad multiplet (9H), 1.25, broad singlet, (24H), 1.32-1.47, broad (3H), 2.20-2.73, multiplet, (6H) 3.55, t, 8=Hz, (2H), 3.81, multiplet, (1H).

- ^{13}C NMR (101 MHz, cdcl3) δ 14.28, 22.84, 23.23, 25.51, 29.49, 29.77, 32.06, 47.46, 52.99, 67.22. (Some signals broad, not enough resolution to see detect all carbons)

NMR data for dialkylamine Azetidinium Salts molecules (Closed form)

- 3-hydroxy-1-methyl-1-undecylazetidin-1-ium chloride [C_{11}-N-C_1-Prop-2-OH]

  ^1H NMR (400 MHz, DMSO) δ 0.81, t, J=8Hz, (3H), 1.21-1.41, m (14H), 2.22-2.42, m, (2H), 3.48-3.57, m, (2H), 3.60-3.73, m, (6H), 4.98, d, J=9 Hz, (1H).

- 1-hexyl-3-hydroxy-1-undecylazetidin-1-ium chloride [C_{11}-N-C_3-Prop-2-OH]

  ^1H NMR (400 MHz, DMSO) δ 0.85, t, J=8Hz, (3H), 0.89, t, J=8Hz, (3H), 1.21m (16H), 1.51, broad, (4H), 3.18-3.37, m, (4H), 4.02, d, J=8Hz, (2H), 4.43, dd, J=2 and 8 Hz, (2H), 4.61, d, J=4Hz, (1H)

- 1-hexyl-3-hydroxy-1-undecylazetidin-1-ium chloride [C_{11}-N-C_6-Prop-2-OH]

  ^1H NMR (400 MHz, DMSO) δ 0.86, t, J=8Hz, (6H), 1.21-1.26 m (22H), 1.51, broad, (4H), 3.20-3.37, m, (4H), 4.06, d, J=8Hz, (2H), 4.42, dd, J=2 and 8 Hz, (2H), 4.61, d, J=4Hz, (1H)

- 1-hydroxy-1-nonyl-1-propylazetidin-1-ium chloride [C_9-N-C_3-Prop-2-OH]

  ^1H NMR (400 MHz, CDCl3) δ 0.85 (dt, J = 7.2, 3.7 Hz, 6H), 1.16 – 1.54 (m, 16H), 2.23 – 2.80 (m, 6H), 4.20 – 4.30 (m, 1H), 4.46 (td, J = 8.2, 2.9 Hz, 1H), 4.63 – 4.74 (m, 1H).

- 1-(2-ethylhexyl)-3-hydroxy-1-undecylazetidin-1-ium chloride [C_{11}-N-$C_{6(2Et)}$-Prop-2-OH]

^1H NMR (400 MHz, DMSO) δ 0.86, broad, (9H), 1.21-1.26 m (24H), 1.55, broad, (3H), 3.20-3.37, m, (4H), 4.06, d, J=8Hz, (1H), 4.15, d, J=8Hz, (1H), 4.37, d, J=8Hz, (1H), 4.52, d, J=8 Hz, (1H), 4.67, d, J=4Hz, (1H)

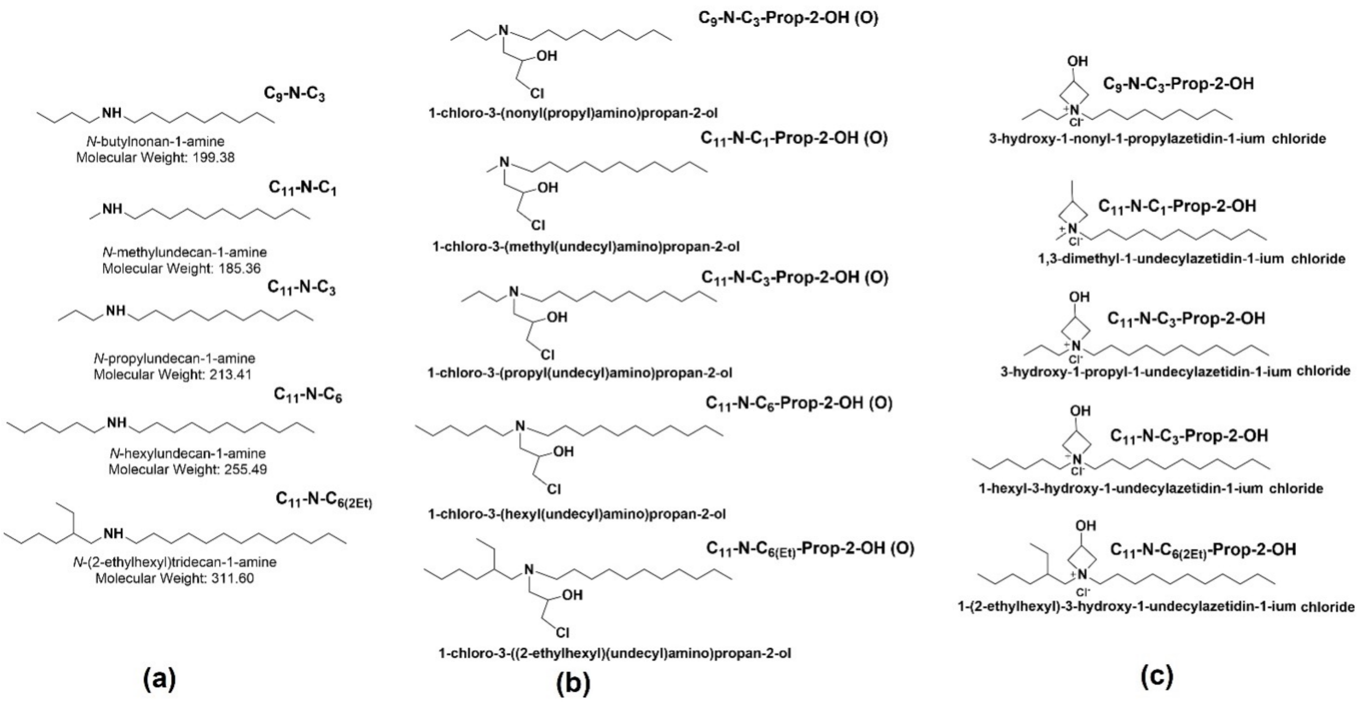

Figure SI 3: Chemical structures and nomenclature of dialkyl amine and dialkyl azetidinium molecules: (a) dialkylamines, (b) dialkylamine azetidinium salts (open), (c) dialkylamine azetidinium salts (closed).

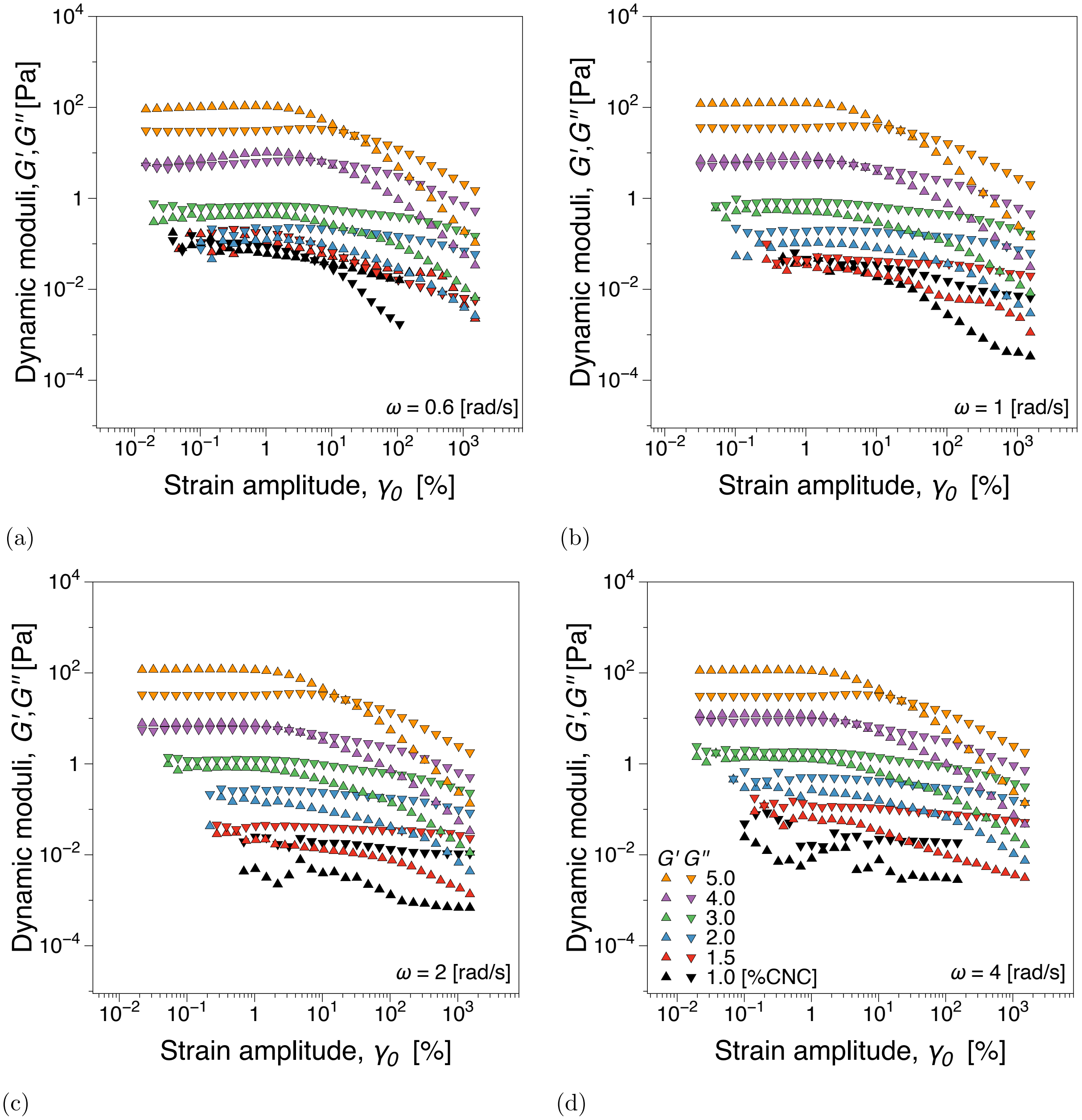

Figure SI 4: Dynamic storage (G') and Loss moduli (G'') for all concentrations of CNC-OSO_3H from strain sweep tests for ω = (a) 0.6, (b) 1, (c) 2 and (d) 4 rad/s.

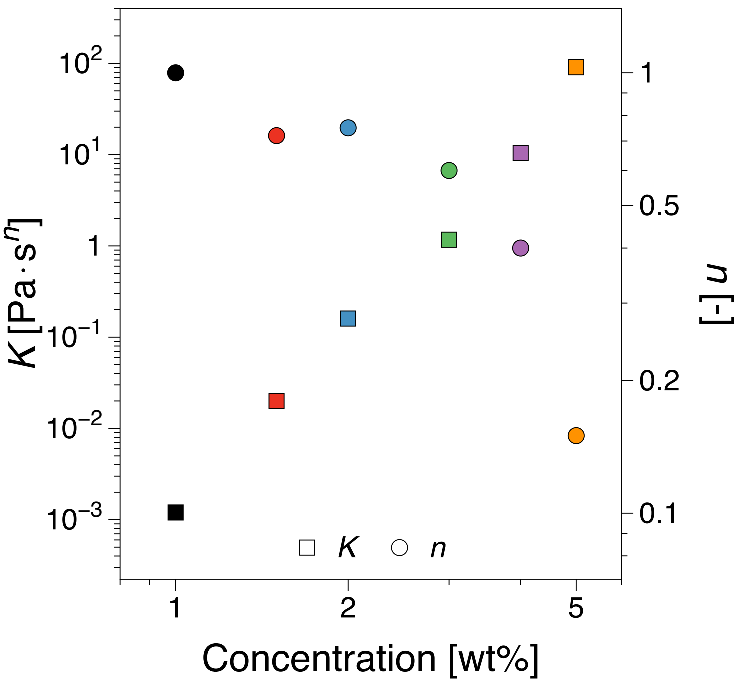

Figure SI 5: Power law model parameters, the consistency index K and the flow index n, from fittings of the complex viscosity functions in Fig. 2 (a).

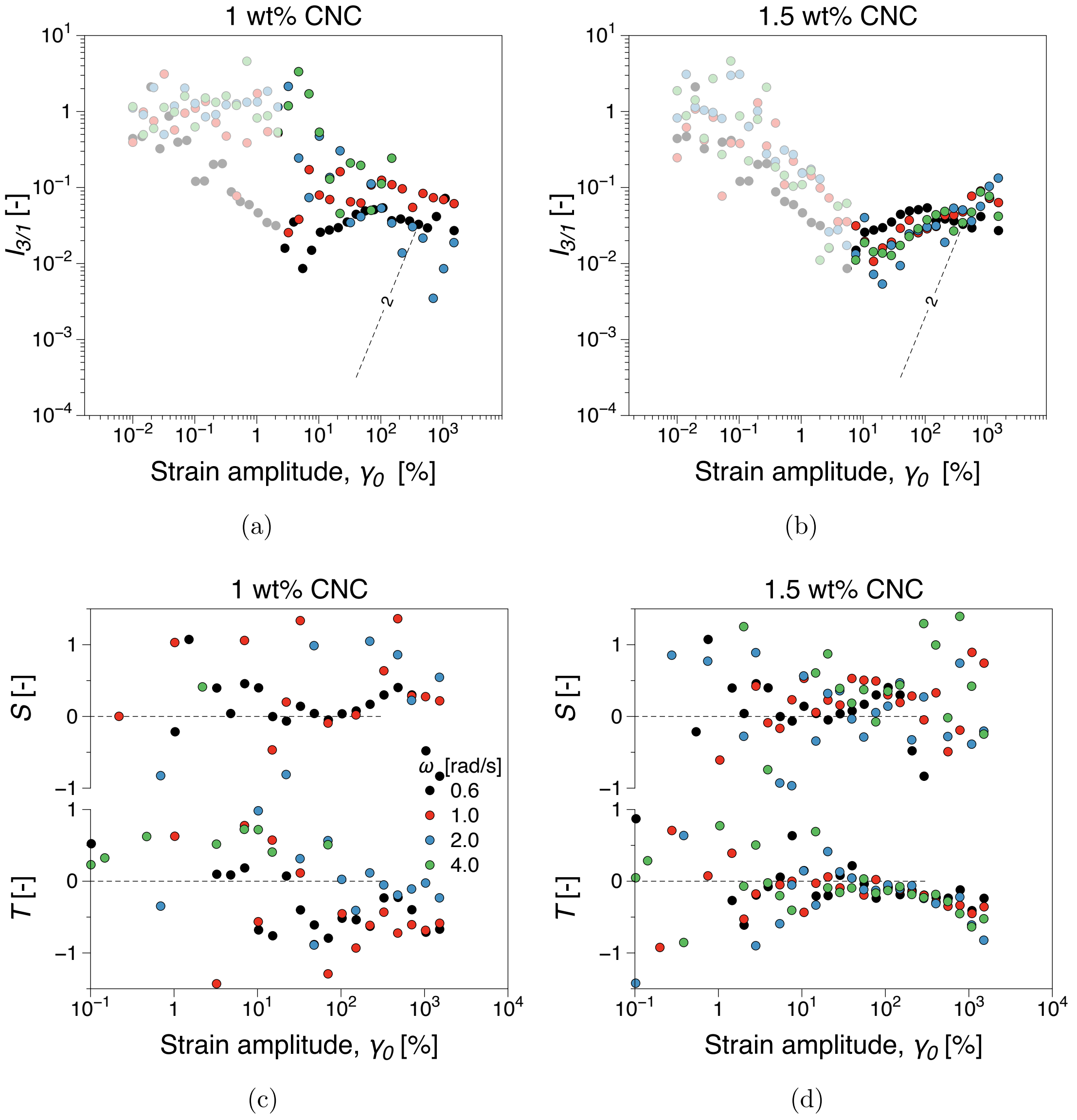

Figure SI 6: The nonlinear material response represented by the third relative higher harmonic, $I_{3/1}$, from dynamic strain sweeps for different concentrations of CNC-OSO_3H suspensions (a) 1, (b) 1.5 wt% and the stress decomposition parameters, strain-stiffening, S, and shear-thickening, T (c) 1, (d) 1.5 wt%.

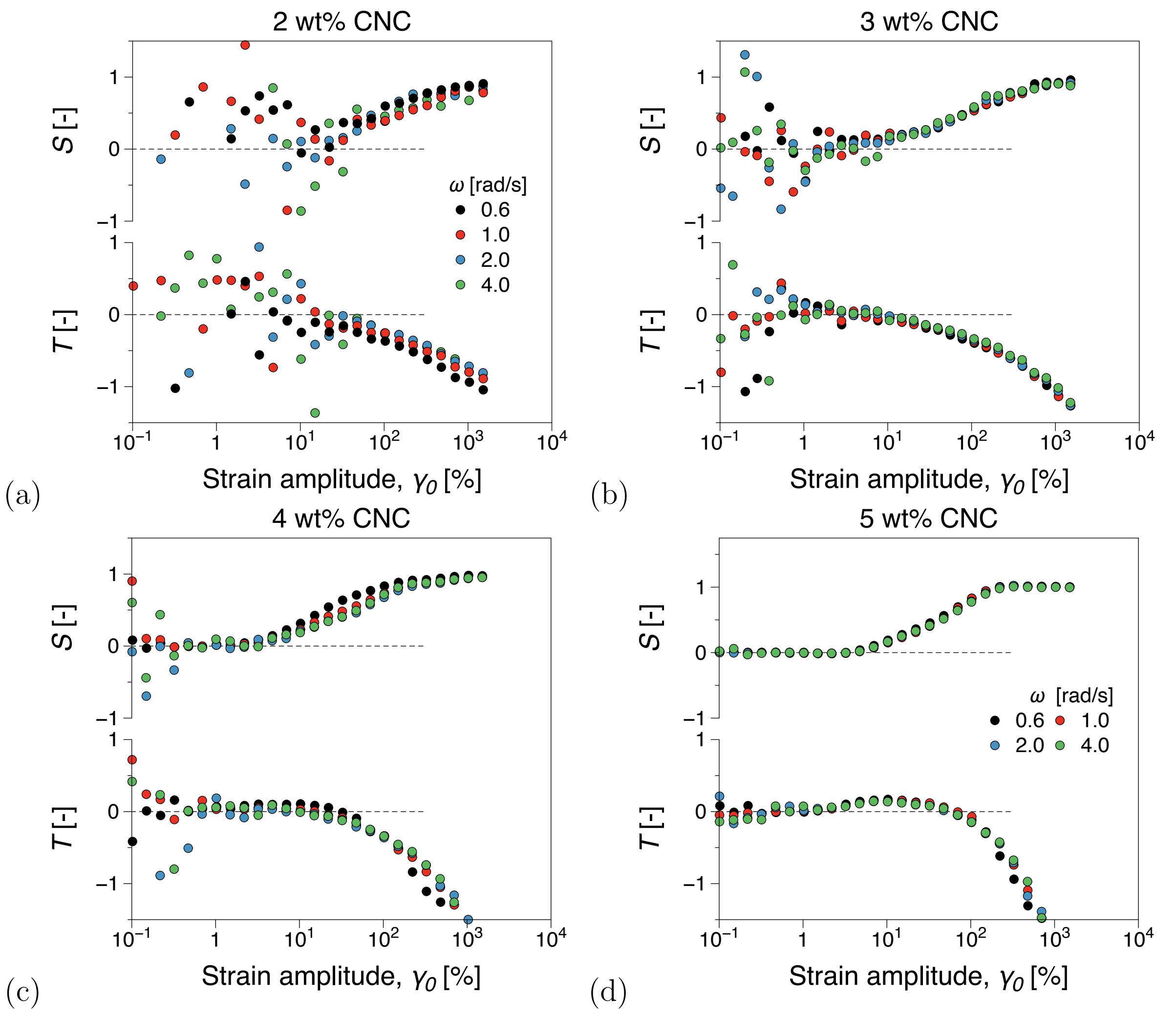

Figure SI 7: S, T parameters for pristine CNC: (a) 2 wt%, (b) 3 wt%, (c) 4 wt%, (d) 5 wt% from strain sweep measurements.

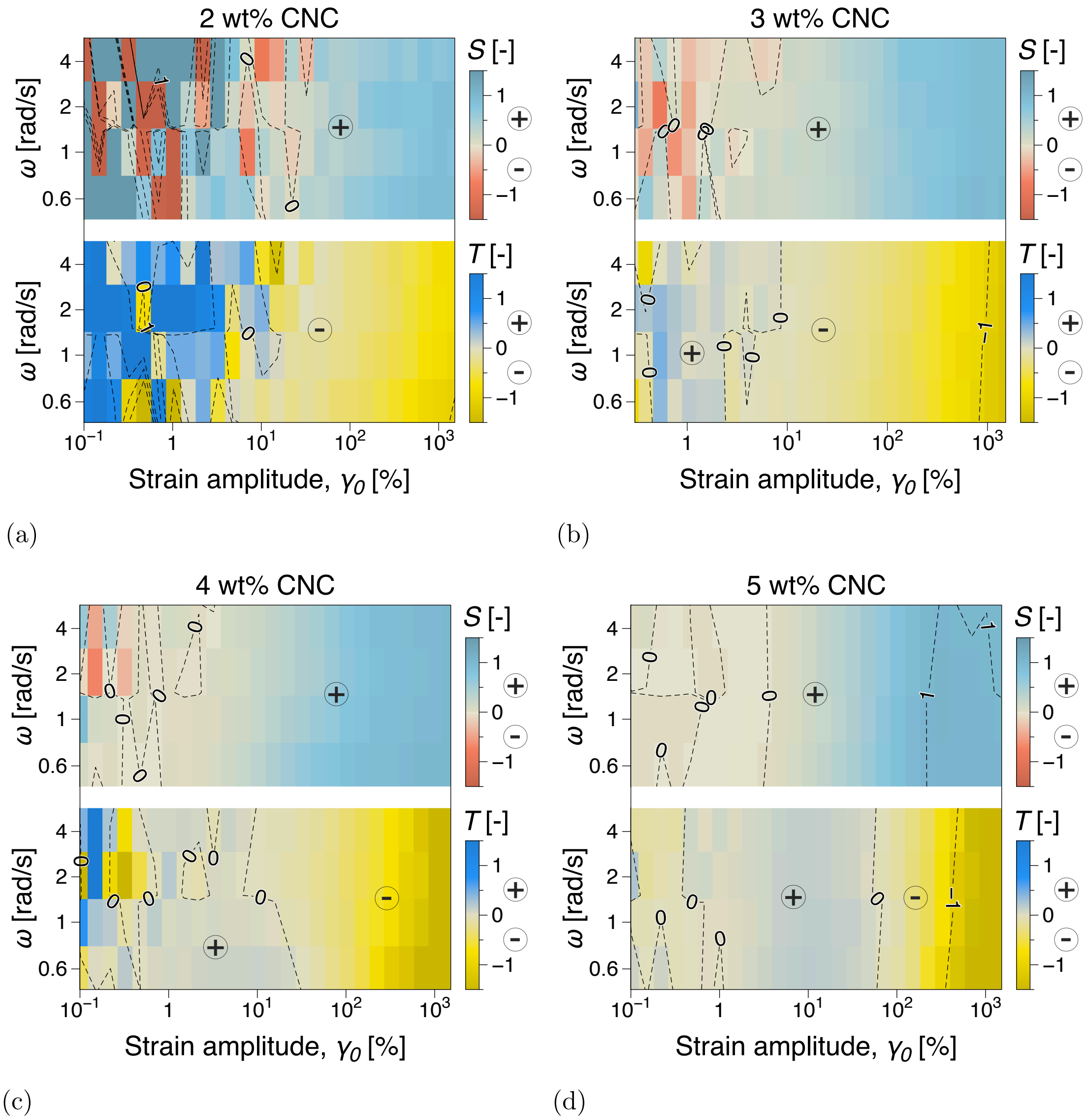

Figure SI 8: Scalar plots of strain-stiffening, S, and shear-thickening, T, nonlinear material parameters for $\omega = 0.6, 1, 2, 4$ rad/s: (a) 2, (b) 3, (c) 4, (d) 5 wt%. The color intensity represents the S, T magnitudes and sign according to the color ramps. To help identifying especially changes in sign and the transition to nonlinear material response, iso-S, T Contours lines for $S, T = -1, 0, 1$ are overlaid as dotted lines and '+' and '-' sign is indicate a change in sign in the nonlinear region.

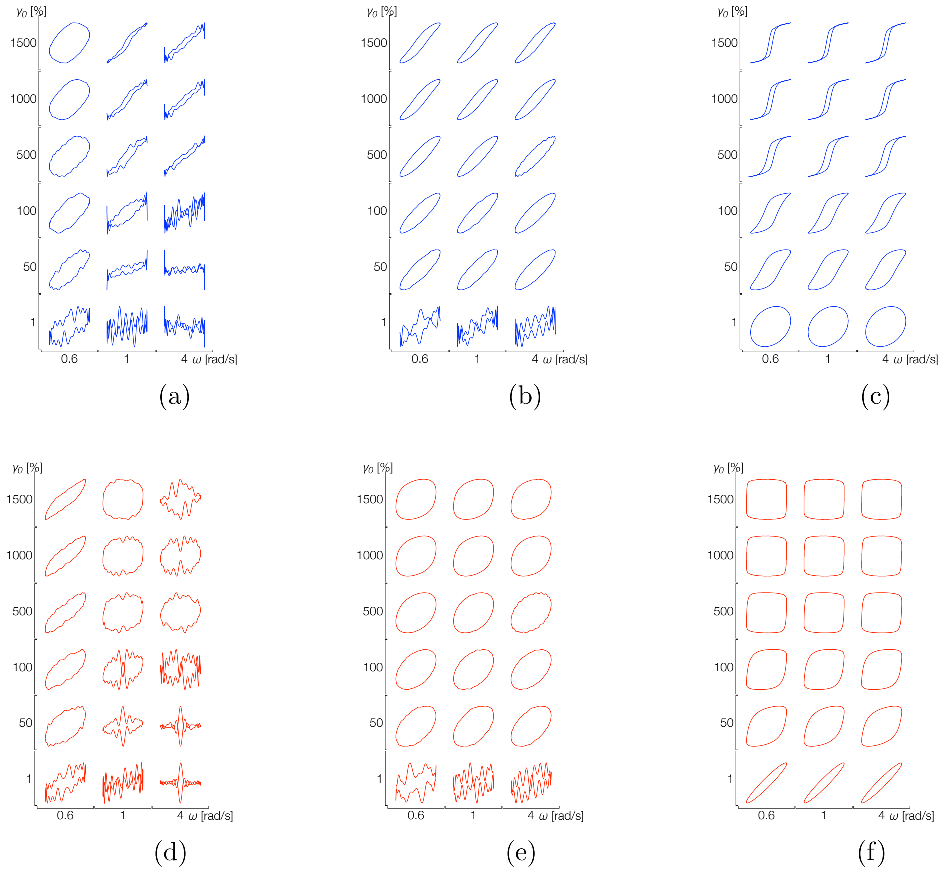

Figure SI 9: Lissajous-Bowditch (LB) diagrams for CNC-OSO_3H suspensions for selected $\omega = 0.6, 1, 4$ rad/s for (a,d) 1 wt%, (b,e) 1.5 wt%, (c,f) 5 wt% compiled as Pipkin diagrams. Top row, (a-c) represent the elastic LB diagrams and bottom row, (d-f) represent the viscous LB diagrams.

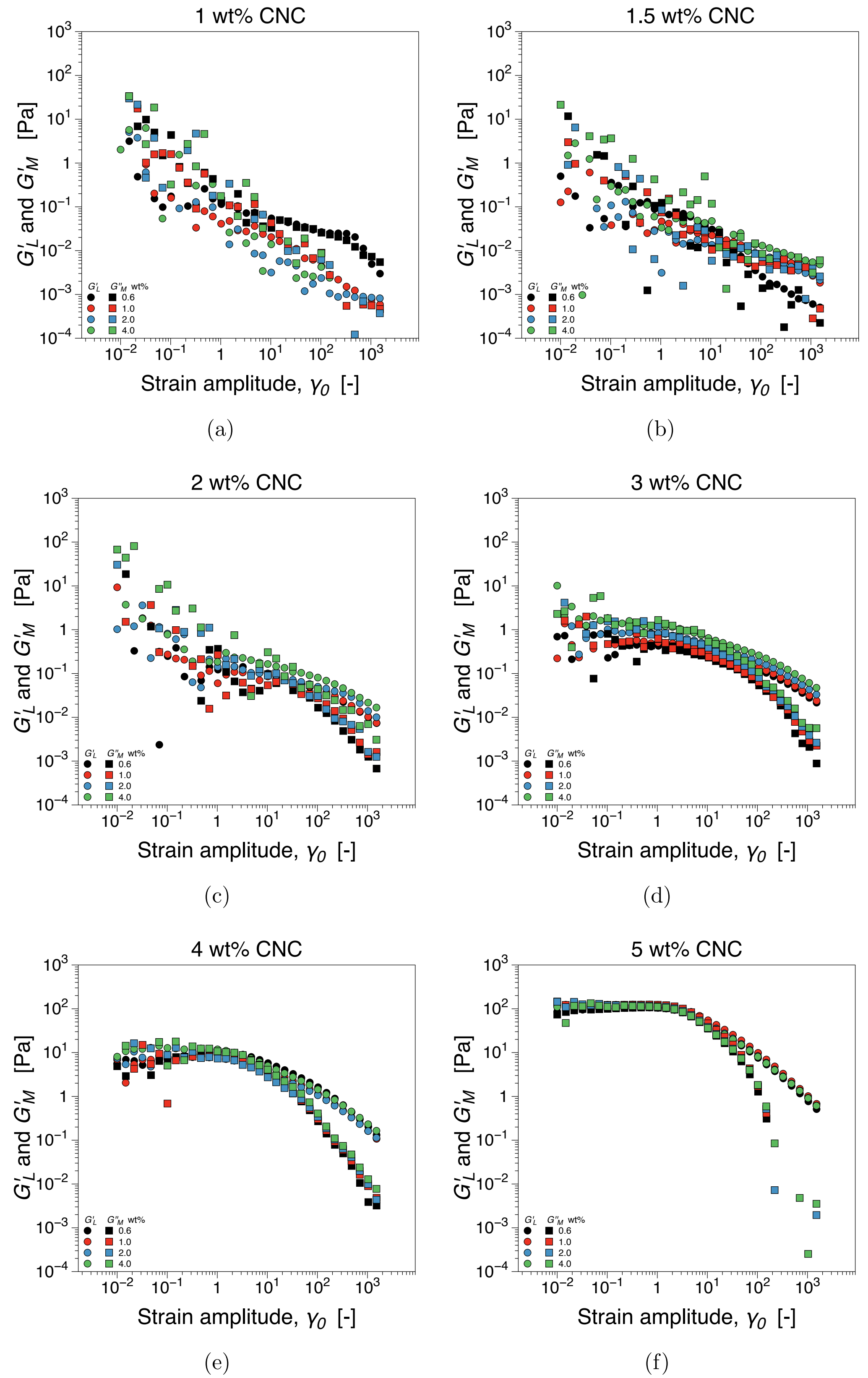

Figure SI 10: Minimum strain rate G'_L and large strain rate G'_M moduli, nonlinear material parameters for $\omega = 0.6, 1, 2, 4$ rad/s: (a) 1 wt%, (b) 1.5 wt%, (c) 2 wt%, (d) 3 wt%, (e) 4 wt%, (f) 5 wt%.

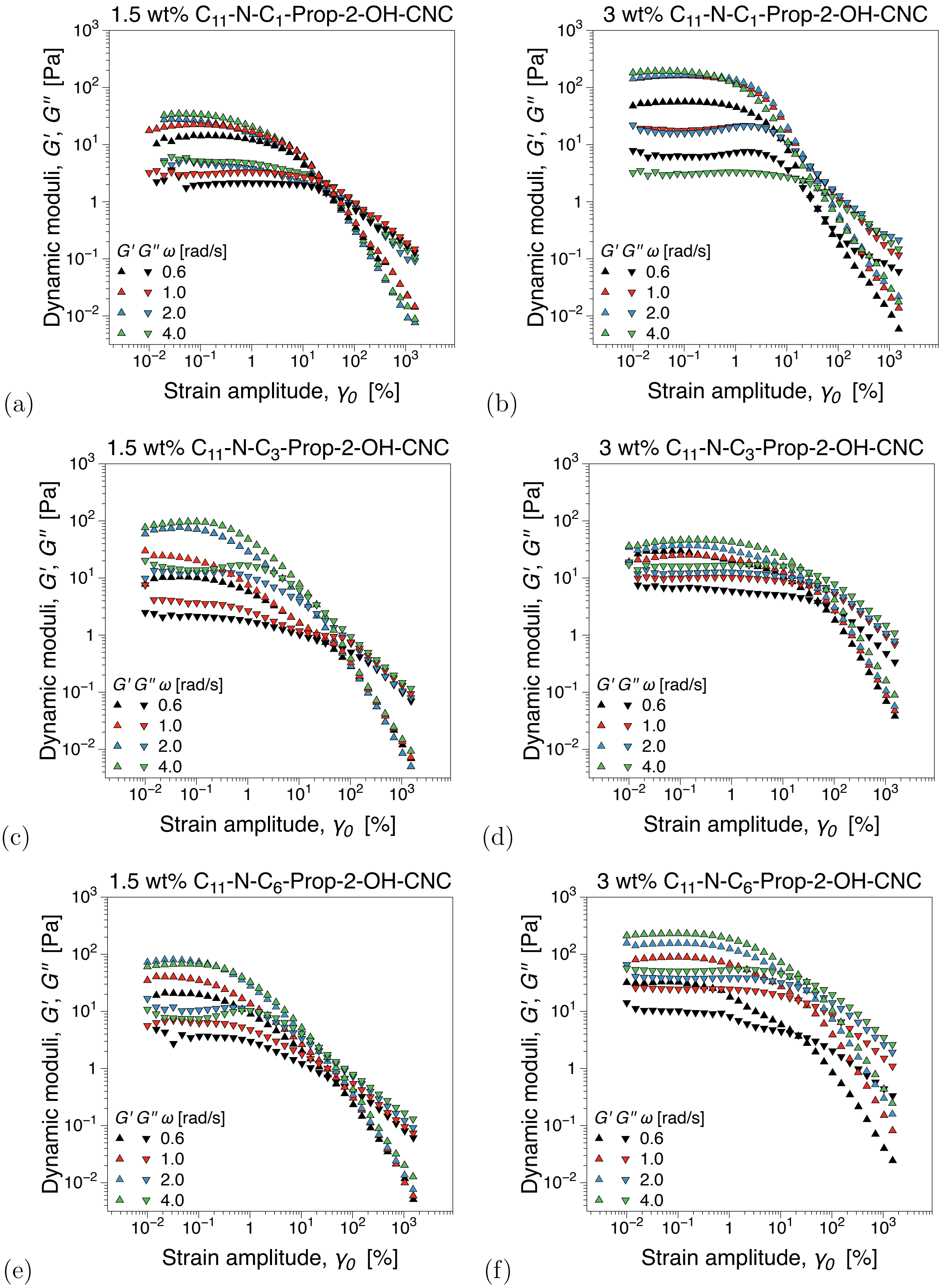

Figure SI 11: (continued)

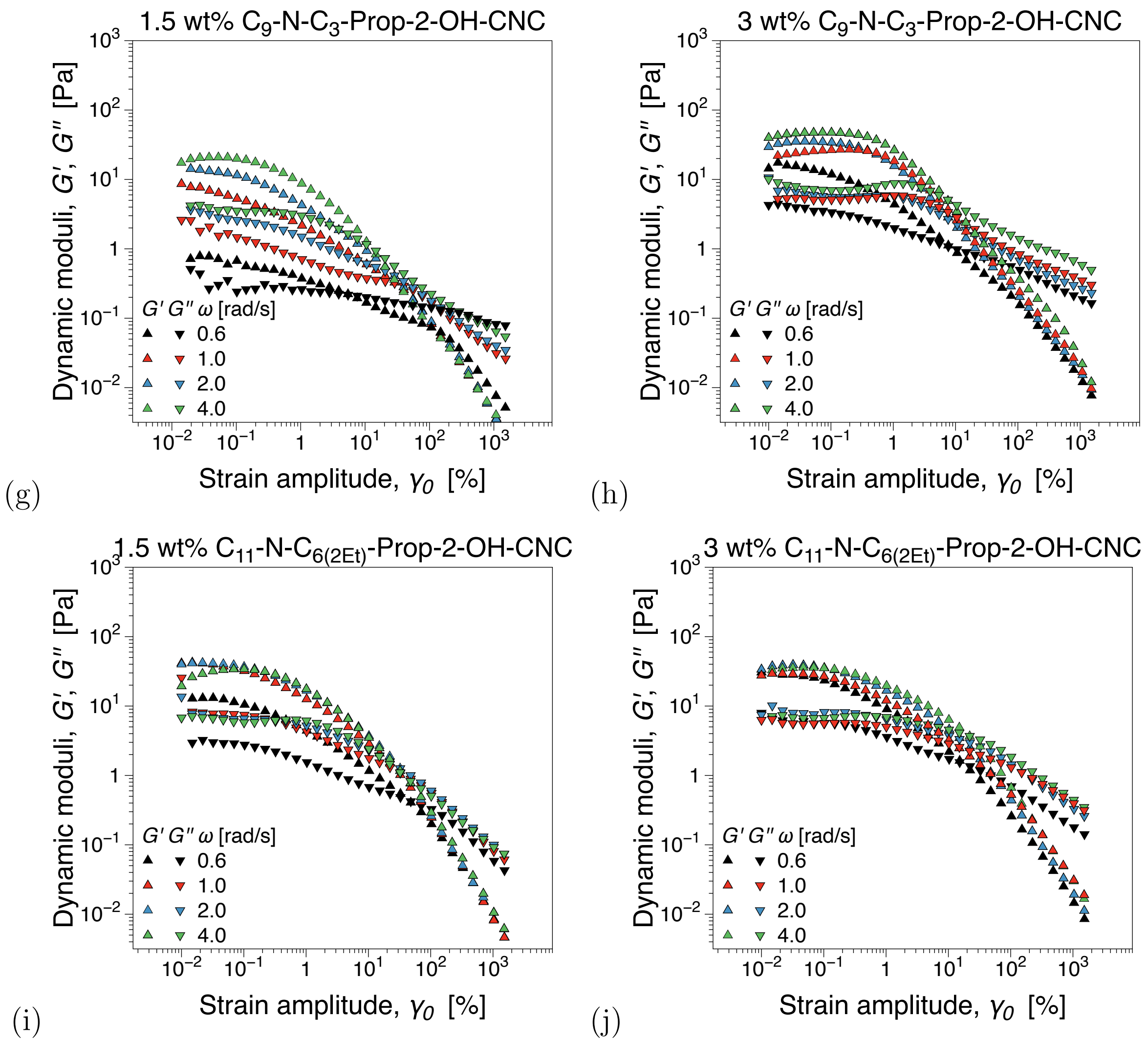

Figure SI 11: Dynamic Storage (G') and Loss moduli (G'') for branched: 11-N-m-Prop-2-OH-CNC, $m = 1, 3, 6$, substituents: (a) $m = 1$ 1.5 wt%, (b) $m = 1$ 3 wt%, (c) $m = 3$ 1.5 wt%, (d) $m = 3$ 3 wt%, and (e) $m = 6$ 1.5 wt% (f) $m = 6$ 3 wt%, 9-N-3-Prop-2-OH- (g) 1.5 wt%, (h) 3 wt% and 11-N-6(2Et)-Prop-2-OH-CNC (i) 1.5wt% and (j) 3 wt% from strain sweep measurements.

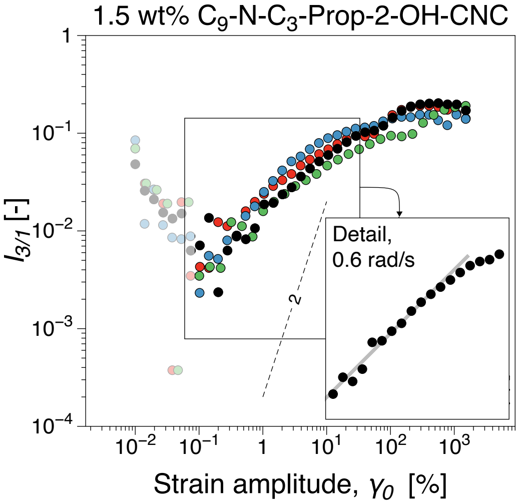

(a)

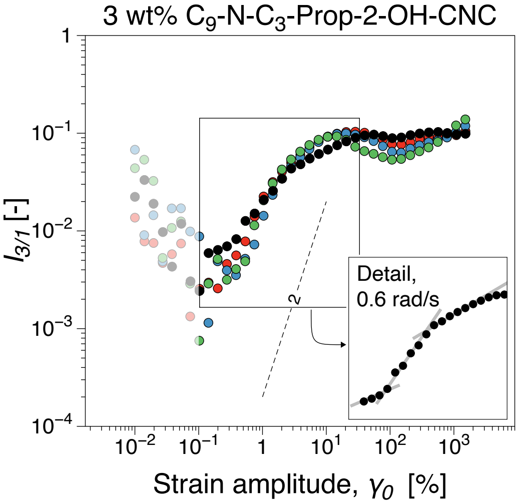

(b)

Figure SI 12: The nonlinear material response represented by the third relative higher harmonic, $I_{3/1}$, from dynamic strain sweeps for different concentrations of 9-N-3-Prop-2-OH-CNC suspensions a) 1.5, b) 3 wt% from strain sweep measurements.

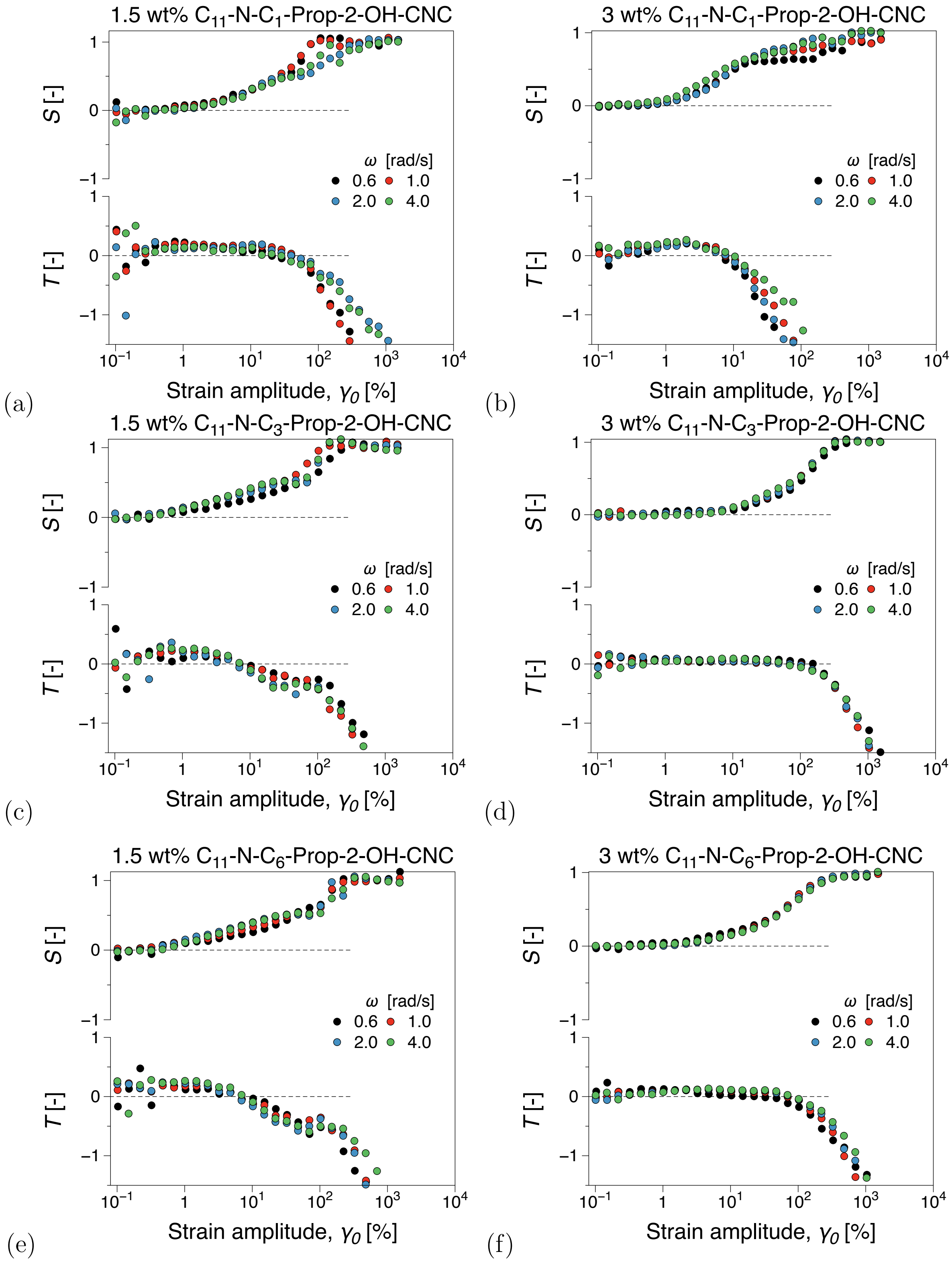

Figure SI 13: (continued)

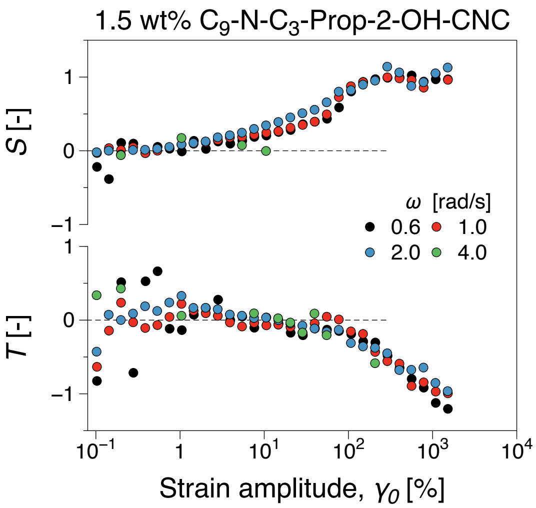

(g)

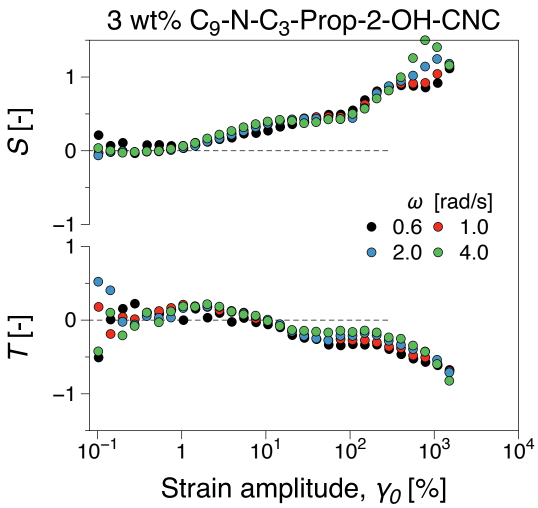

(h)

Figure SI 13: S, T parameters for branched: C_{11}-N-m-Prop-2-OH-CNC, $m = 1, 3, 6$, substituents: (a) $m = 1$ 1.5 wt%, (b) $m = 1$ 3 wt%, (c) $m = 3$ 1.5 wt%, (d) $m = 3$ 3 wt%, (e) $m = 6$ 1.5 wt% (f) $m = 6$ 3 wt%, and C_9-N-C_3-Prop-2-OH-CNC (g) 1.5 wt%, (h) 3 wt% from strain sweep measurements.

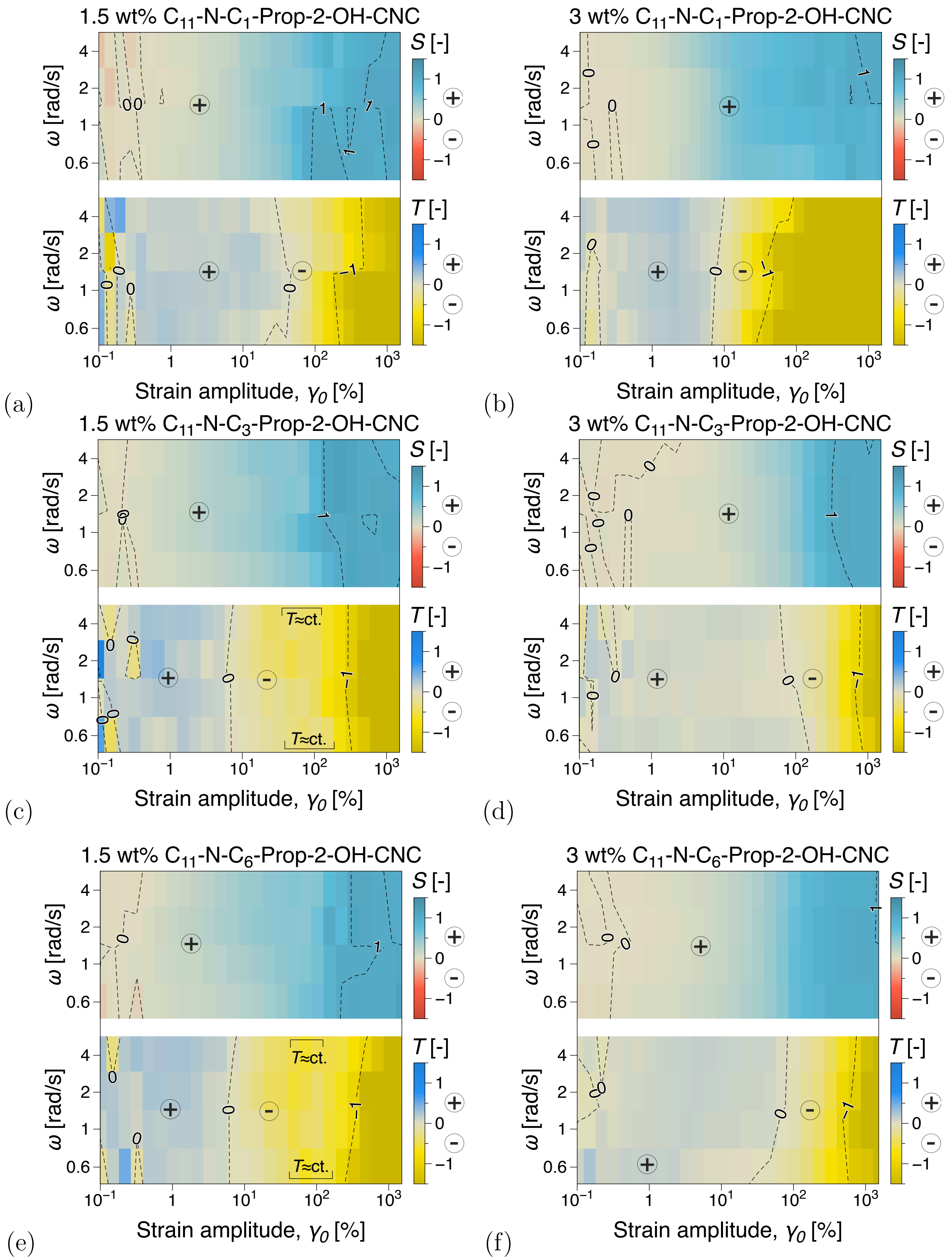

Figure SI 14: S, T parameters for branched: C_{11}-N-m-Prop-2-OH-CNC, $m = 1, 3, 6$, substituents: (a) $m = 1$ 1.5 wt%, (b) $m = 1$ 3 wt%, (c) $m = 3$ 1.5 wt%, (d) $m = 3$ 3 wt%, (e) $m = 6$ 1.5 wt% (f) $m = 6$ 3 wt% from strain sweep measurements. More details about the colormaps can be found in the caption of Fig. 8.

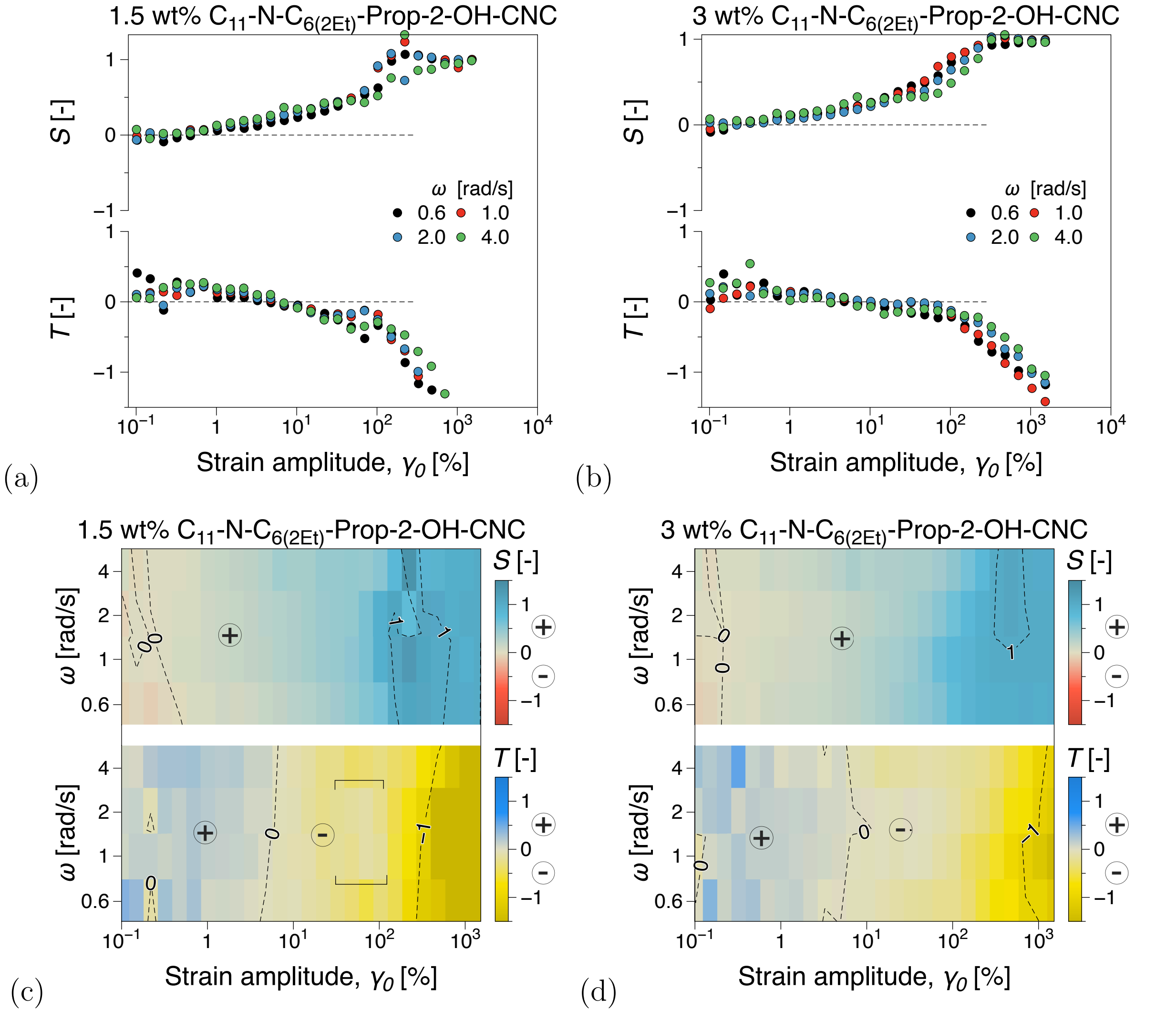

Figure SI 15: S, T parameters for branched-on-branch structure: (a) 1.5 wt%, (b) 3 wt%, visualization as the colormaps: (c) 1.5 wt%, (d) 3 wt%.

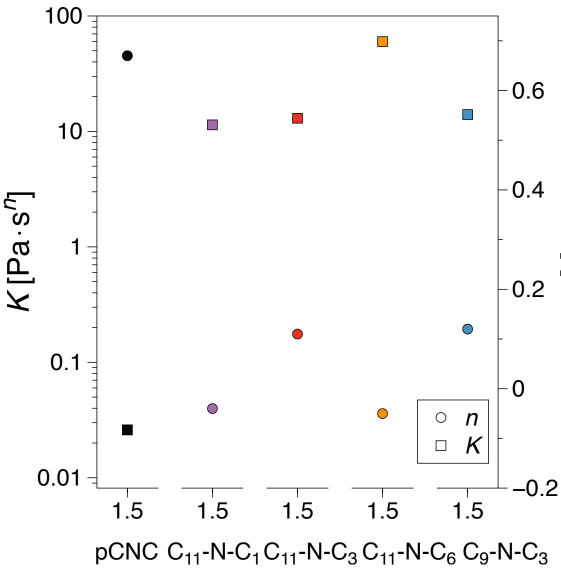

(a)

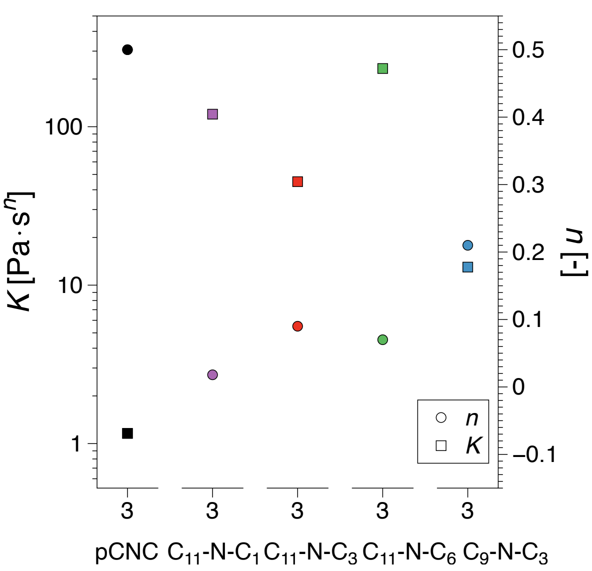

(b)

Figure SI 16: Power law model parameters, (a) the consistency index K and (b) the flow index n, from fittings of the complex viscosity functions in Fig. 7.

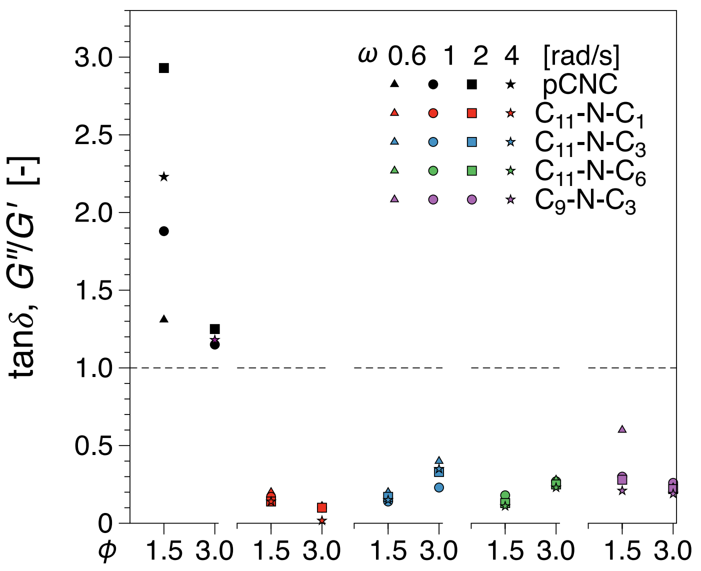

Figure SI 17: Loss tangent, $\tan\delta = G''/G'$, from linear viscoelastic strain sweep measurements as function of branched linkers for both investigated concentrations for all applied angular frequencies.

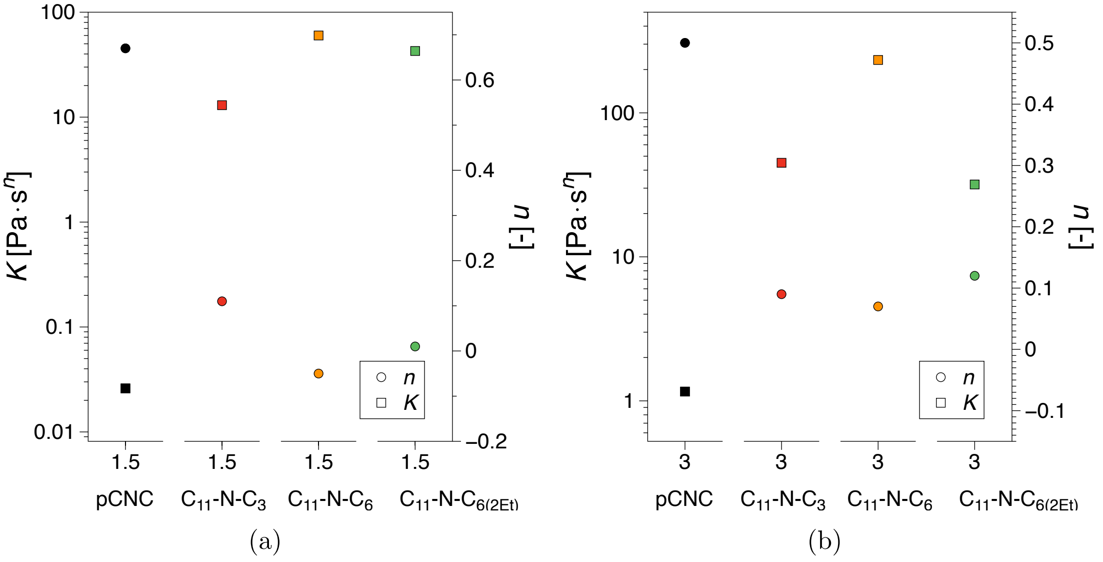

Figure SI 18: Power law model parameters, the consistency index K and the flow index n, from fittings of the complex viscosity functions in Fig. 9, (a) 1.5 wt%, (b) 3 wt% modified CNCs.

References

(1) Lützenkirchen, J.; Preočanin, T.; Kovačević, D.; Tomišić, V.; Lövgren, L.; Kallay, N. Potentiometric titrations as a tool for surface charge determination. *Croatica chemica acta* **2012**, *85*, 391–417.

(2) Wojno, S.; Sonker, A.; Cooper, S.; Gargb, M.; Rigdahl, M.; Linares, M.; Zozoulenko, I.; Kádár, R.; Westman, G. Tuning rheological behavior of cellulose nanocrystalline dispersions by conjugation of symmetric and asymmetric dialkylamine groups. *In review* **2023**,
